# Structural mechanism of a drug-binding process involving a large conformational change of the protein target

**DOI:** 10.1038/s41467-023-36956-5

**Published:** 2023-04-05

**Authors:** Pelin Ayaz, Agatha Lyczek, YiTing Paung, Victoria R. Mingione, Roxana E. Iacob, Parker W. de Waal, John R. Engen, Markus A. Seeliger, Yibing Shan, David E. Shaw

**Affiliations:** 1 D. E. Shaw Research, New York, NY 10036 USA; 2 Department of Pharmacological Sciences, Stony Brook University School of Medicine, Stony Brook, NY 11794-8651 USA; 3 Department of Chemistry and Chemical Biology, Northeastern University, Boston, MA 02115 USA; 4 Department of Biochemistry and Molecular Biophysics, Columbia University, New York, NY 10032 USA; 5 Present Address: Relay Therapeutics, 399 Binney St., Cambridge, MA 02139 USA

**Keywords:** Kinases, Computational biology and bioinformatics, Structural biology, Cancer

## Abstract

Proteins often undergo large conformational changes when binding small molecules, but atomic-level descriptions of such events have been elusive. Here, we report unguided molecular dynamics simulations of Abl kinase binding to the cancer drug imatinib. In the simulations, imatinib first selectively engages Abl kinase in its autoinhibitory conformation. Consistent with inferences drawn from previous experimental studies, imatinib then induces a large conformational change of the protein to reach a bound complex that closely resembles published crystal structures. Moreover, the simulations reveal a surprising local structural instability in the C-terminal lobe of Abl kinase during binding. The unstable region includes a number of residues that, when mutated, confer imatinib resistance by an unknown mechanism. Based on the simulations, NMR spectra, hydrogen-deuterium exchange measurements, and thermostability measurements and estimates, we suggest that these mutations confer imatinib resistance by exacerbating structural instability in the C-terminal lobe, rendering the imatinib-bound state energetically unfavorable.

## Abstract (web summary)

Atomic-level descriptions of protein–small molecule binding processes that involve a large conformational change of the protein have been elusive. Here, the authors report unguided molecular dynamics simulations of such a process—Abl kinase binding the cancer drug imatinib.

## Introduction

Structural studies indicate that many proteins undergo large conformational changes when they bind to small-molecule ligands, yet achieving a detailed understanding of these large changes remains a challenge. Crystal structures, for example, are in some cases available for the unbound and bound states of a protein, but they do not provide an atomic-level view of the entire pathway. Molecular dynamics (MD) simulations, on the other hand, can provide a continuous view of binding^1,2^, but these studies have generally focused on binding events characterized by relatively modest changes in protein conformation. By way of example, we previously reported unbiased MD simulations of the drug dasatinib binding to Src kinase^2^, a process that occurs without any substantial conformational changes of the protein^3^. A well-known example of a process that is known to require a substantial conformational change (involving 20 residues with displacements averaging >15 Å) is the cancer drug imatinib binding to its target, Abelson tyrosine kinase (Abl). Previously, some intermediate conformations in this binding process were inferred from simulations starting from imatinib bound with Abl or the Abl homolog Src (using biasing forces to induce imatinib unbinding and binding)^4,5^, but simulations of the binding process without using biasing forces have not been reported.

In 2001, imatinib was approved for the treatment of chronic myeloid leukemia (CML), making it the first small-molecule kinase inhibitor to receive FDA approval as a cancer therapy^6,7^. CML results from expression of the constitutively active tyrosine kinase Bcr-Abl, a fusion of the breakpoint cluster region (Bcr) protein and the kinase domain of Abl (Abl kinase). Imatinib binds and inhibits the kinase activity of Bcr-Abl, and imatinib therapy can give CML patients a normal life expectancy if provided at an early stage of the disease. Unfortunately, patients whose disease is more advanced at the time of diagnosis often develop mutations in Abl kinase that render them resistant to imatinib. These mutations are almost exclusively located in the kinase domain^8^, suggesting that imatinib binding is little affected by the other domains, and resistance predominantly arises from altered dynamics of the kinase domain. Achieving a more detailed understanding of Abl-imatinib binding could potentially suggest strategies to combat imatinib resistance or inform new directions in kinase-targeting drug discovery.

Abl kinase consists of a smaller N-terminal lobe and a larger C-terminal lobe (the N- and C-lobes, respectively), with an ATP-binding site located between them^9^. The N-lobe consists of five β-strands and a helix (the αC helix), whereas the C-lobe consists of multiple helices and includes an activation loop (A-loop or AL) and a myristoyl-binding site that is important for Abl regulation (Supplementary Fig. 1A)^10^. Within the ATP-binding site, there is a highly conserved sequence of three residues—the Asp-Phe-Gly (DFG) motif—that plays a critical role in defining the active and autoinhibitory states of the kinase. In the catalytically active state, the DFG motif adopts a “DFG-in” conformation, which allows ATP binding, and the A-loop adopts an “open” conformation, which allows substrate binding; we will refer to this as the DFG-in/AL-open conformation. In the autoinhibitory conformation, the DFG motif is “flipped” to adopt a “DFG-out” conformation, while the A-loop remains open (the DFG-out/AL-open conformation). These conformational states and their relative populations have been systematically characterized using NMR^11^. Imatinib binding at the ATP-binding site causes Abl to undergo a significant conformational change in which the A-loop adopts a closed conformation that precludes substrate binding, while the DFG motif is locked in the DFG-out conformation (i.e., the DFG-out/AL-closed (inactive) conformation; Supplementary Fig. 1B)^12,13^. This conformation is distinct from the active conformation to which dasatinib binds (Supplementary Fig. 1B).

Here, we present unbiased MD simulations of the process of Abl-imatinib binding. These simulations capture the conformational change from the DFG-in/AL-open conformation to the DFG-out/AL-closed conformation, and successfully reproduce the known Abl-imatinib complex (Supplementary Movie 1). The main features of the binding mechanism observed in these simulations are in agreement with conclusions drawn from previous kinetic experiments showing that imatinib binding is a two-step process^3,14^. The simulations are also in agreement with a nuclear magnetic resonance (NMR) study showing that the binding process involves a hybrid mechanism of conformational selection and induced fit; it begins with a conformational selection step (in which Abl kinase adopts a DFG-out conformation to which imatinib binds), followed by a large conformational rearrangement that is induced by the binding of imatinib (an induced-fit mechanism)^15^. In addition to providing confirmation of the binding scheme inferred from experiments, the simulations provide a detailed description of key states that were not elucidated by the NMR studies, and also of transitions in the binding process.

An unexpected observation from the simulations was that the conformational change associated with imatinib binding involves local structural destabilization at the core of the C-lobe, where energetically unfavorable conformations are adopted in which native residue-residue contacts are disrupted. This instability is most pronounced during the conformational change, but also remains after the conformational change (i.e., in the imatinib-bound state), though to a lesser degree. Interestingly, there are a number of residues clustered in this destabilized region of the C-lobe that confer imatinib resistance when mutated in patients^8^, and it has been unclear how these mutations, distal from the imatinib-binding site, confer resistance. The results from the simulations, together with our experimental results, suggest that these mutations impair the structural stability of Abl kinase, and the additional instability incurred at the C-lobe with imatinib binding renders the imatinib-bound form energetically unfavorable.

## Results

### Imatinib binding starts with conformational selection for the autoinhibitory state

**Fig. 1:**
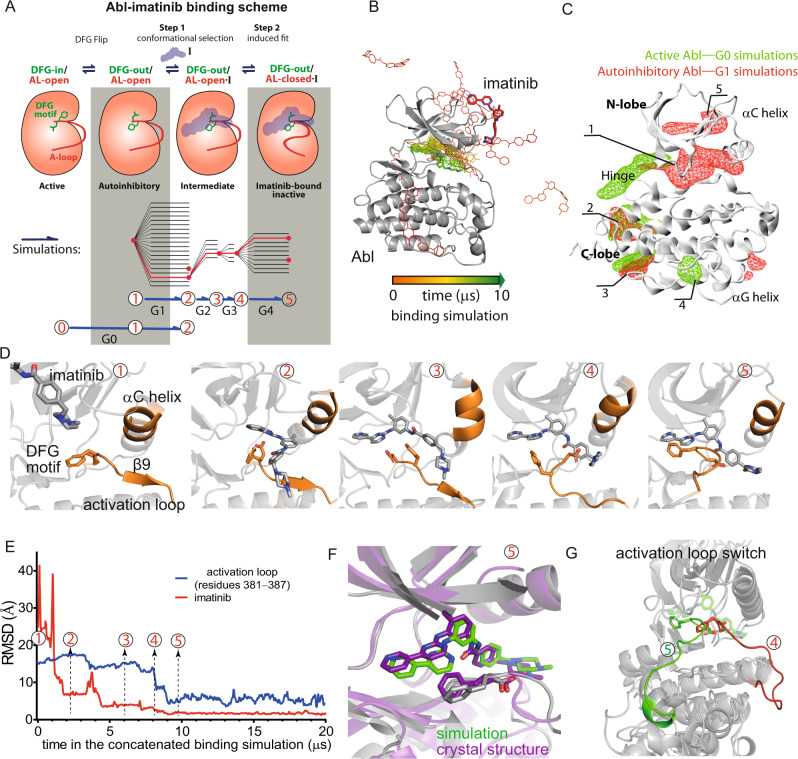
Simulations of Abl-imatinib binding. **A** Top: A diagram describing the scheme of Abl-imatinib binding, starting with the spontaneous DFG flip of the apo kinase with an open activation loop (AL) (i.e., from the DFG-in/AL-open to the DFG-out/AL-open conformation). A binding intermediate is then formed with imatinib (DFG-out/AL-open·I), which resolves to the native imatinib-bound state (DFG-out/AL-closed·I). Bottom: The five generations of simulations (G0–G4) that together span the binding process. Each gray line represents an individual simulation, and each blue arrow represents a group of simulations. The connected red lines represent a set of four sequential simulations that, when concatenated, describe the entire binding process (together, we refer to these four simulations as the “concatenated binding simulation”). The red dots represent simulation snapshots that are representative of conformational states denoted by the corresponding circled numbers below (this labeling scheme is also used in the other panels). States 0, 1, 3, and 5 respectively correspond to the four states illustrated in the binding diagram (these are the more stable states), while States 2 and 4 represent the more transient states in the binding process. **B** The imatinib molecule moving from the free state to the bound state in the concatenated binding simulation; the native pose is reached in ~10 μs. **C** Imatinib occupancy derived from all G0 simulations (green) and derived from all G1 simulations (red). The imatinib molecules primarily interacted with Abl kinase at the following five labeled regions: (1) the ATP-binding site and its extension between the N- and the C-lobes; (2) the interface with the SH2 domain used in Abl autoinhibition; (3) the myristoyl-binding site; (4) the αG helix region of the C-lobe; and (5) the PIF-binding site of the N-lobe. **D** Snapshots of the imatinib-binding site corresponding to the numbered states from the concatenated binding simulation. **E** Imatinib root-mean-square deviation (RMSD) with respect to the native pose (red) and A-loop RMSD with respect to the closed conformation (blue) as functions of time in the concatenated binding simulation. The five states (States 1–5) are shown. **F** Imatinib-bound pose from the simulation superimposed on the imatinib-bound crystal structure pose (PDB ID: 1OPJ). **G** Starting and final conformations of the A-loop from the concatenated binding simulation.

Figure 1A shows schematic representations of both our simulations (bottom) and the imatinib-binding process (top). For the simulations, each gray line represents an individual simulation, and each blue arrow represents a group of simulations. The connected red lines indicate a set of four sequential simulations that, when concatenated, describe the Abl-imatinib binding process in its entirety (Fig. 1B)—together, we will refer to these four simulations as the “concatenated binding simulation.” (These simulations are described in detail below.) The concatenated binding simulation suggests a binding process (Fig. 1A, top) in which both conformational selection and induced fit mechanisms are crucial. In addition, the simulations showed that binding involved four primary conformational states—the DFG-in/AL-open and DFG-out/AL-open states of the apo kinase, and the DFG-out/AL-open·I (intermediate) and DFG-out/AL-closed·I (native) states of the imatinib-bound kinase—along with two additional, more transient, conformational states. These conformational states are denoted by the circled numbers in Fig. 1A, with States 0, 1, 3, and 5 being the primary states, and States 2 and 4 being the more transient states. The red dots in Fig. 1A indicate simulation-generated conformations representative of one of the denoted states. The states are discussed in detail below.

Initially, it was unclear which conformation of Abl should be used to initiate simulations of Abl-imatinib binding in order to best reflect the binding process that occurs in nature. Kinetic experiments have shown that binding is a two-step process that includes a rate-limiting step of conformational change^3,14^, suggesting that the binding process does not begin from the DFG-out/AL-closed conformation of the native Abl-imatinib complex. Moreover, it is likely that the DFG-out/AL-closed conformation is rarely accessible by Abl in the absence of imatinib, as it has only been captured in crystal structures of Abl bound with imatinib or imatinib analogs. We thus started the imatinib binding simulations from either the DFG-in/AL-open (active) or DFG-out/AL-open (autoinhibitory) conformations of apo Abl kinase (Fig. 1A), but not the DFG-out/AL-closed conformation.

**Fig. 2:**
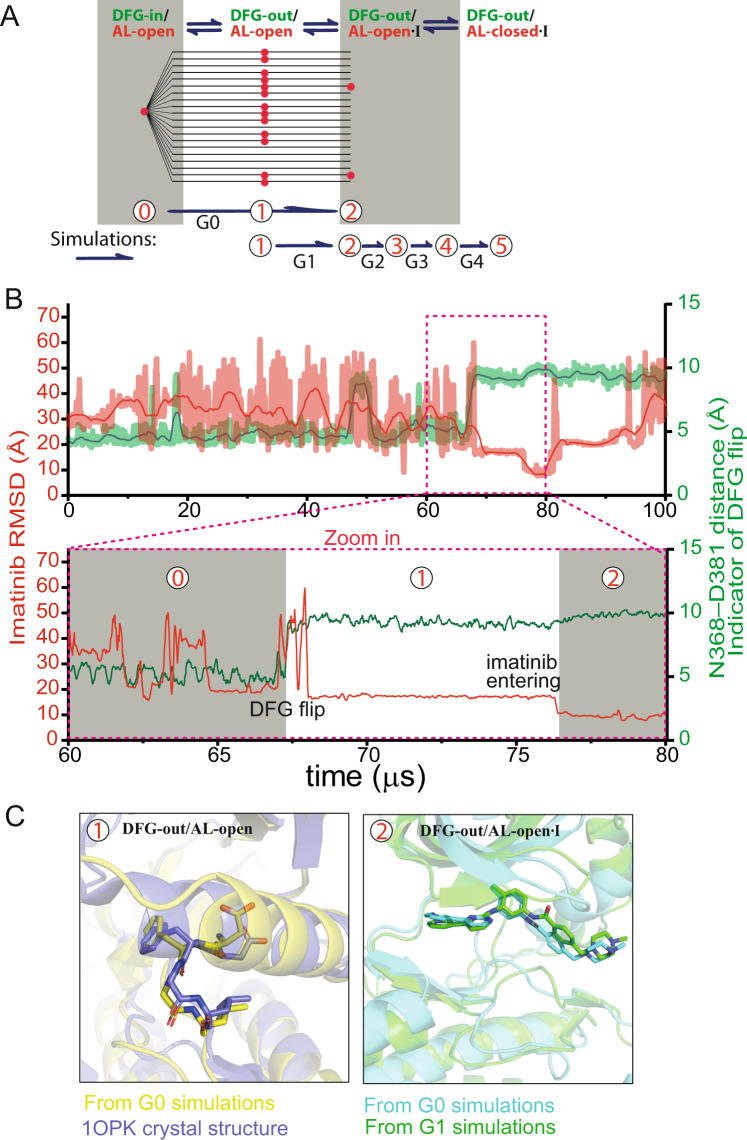
Simulations starting from the active Abl kinase conformation. **A** G0 simulations, which start from the active conformation (State 0). The DFG flip occurred in most of these simulations (i.e., State 1 was reached) and two simulations reached State 2. The binding diagram and G1–G4 simulations are shown and explained in Fig. 1A. **B** Imatinib RMSD with respect to the native pose (red) and Asn368-Asp381 distance indicative of DFG conformation (green) as functions of time in one of the G0 simulations that reached State 2. The DFG flip occurred at ~67 µs. **C** Left: A representative snapshot after the DFG flip in the simulation superimposed on the DFG-out/AL-open crystal structure (PDB ID: 1OPK). Right: A representative snapshot from a G0 simulation superimposed on State 2 from the concatenated binding simulation shown in Fig. 1A.

We first performed 20 simulations (lasting 292 µs in aggregate) in which three imatinib molecules were arbitrarily placed in the vicinity of unphosphorylated Abl kinase in the apo DFG-in/AL-open (active) conformation (which we sometimes refer to, more briefly, as State 0) (see Methods). Asp381 of the DFG motif was protonated because its pKa is high due to the local electrostatic environment (the pKa is estimated to be 6.6 for Apo Abl kinase, indicating a substantial population of Asp381 is protonated at equilibrium^3^). Further, it has been shown that protonation promotes flipping to the DFG-out conformation^3^. In 13 of these 20 simulations (as shown in Fig. 2A), Abl kinase underwent the DFG flip (from the DFG-in to the DFG-out conformation) and arrived at the autoinhibitory DFG-out/AL-open conformation (which we sometimes refer to as State 1). The DFG flips occurred both prior to and after imatinib entering the ATP-binding site, suggesting that the DFG flip was not induced by imatinib, and that recognizing the flipped (i.e., the DFG-out/AL-open) conformation is the likely first step of imatinib binding. Further analysis later confirmed that these simulations captured the very first step of the binding process, and we thus refer to them as Generation 0 (G0) simulations. In these simulations, imatinib interacted non-specifically and transiently with various regions of Abl kinase (Fig. 1C), and there were multiple occurrences of imatinib entering and remaining in the ATP-binding site until the end of the simulation.

We also launched 20 simulations (lasting 152 µs in aggregate) of Abl-imatinib binding starting from State 1, the autoinhibitory DFG-out/AL-open conformation of the apo kinase. These are termed G1 simulations because we concluded that they captured the step of imatinib binding following the DFG flip. Prior to initiating these simulations, three imatinib molecules were arbitrarily placed in the vicinity of Abl kinase. Asp381 was protonated in these simulations as well because its pKa in the DFG-out/AL-open conformation was estimated to be >11^3^. As expected, the autoinhibitory conformation remained stable throughout all 20 simulations. The imatinib molecules primarily interacted with Abl kinase at the following five regions, with residence times of microseconds to tens of microseconds: (1) the ATP-binding site and its extension between the N- and the C-lobes; (2) the interface with the SH2 domain used in Abl autoinhibition^10^; (3) the myristoyl-binding site (Supplementary Fig. 2A); (4) the αG helix region of the C-lobe; and (5) the so-called PIF-binding site^16^ of the N-lobe (Fig. 1C). Intriguingly, in the ATP-binding site, we observed that imatinib adopted a pose that has been seen in a crystal structure of imatinib in complex with Syk kinase (Supplementary Fig. 2B), suggesting that this pose may represent a minor state in Abl-imatinib binding. In contrast to the simulations starting from the active conformation, in which imatinib interactions were limited to the hinge region and did not involve the αC helix, the simulations starting from the autoinhibitory conformation showed imatinib interacting with Abl in a broader region between the two lobes, as in the native imatinib-bound pose (Fig. 1C). We thus reasoned that the G1 simulations captured the step of imatinib binding after the DFG flip.

### Simulations recapitulated the process of Abl-imatinib binding and reproduced the native complex structure

As the G1 simulations suggested that binding begins with imatinib recognizing the autoinhibitory (DFG-out/AL-open) conformation of Abl kinase and entering the ATP-binding site, we next initiated a series of consecutive simulations (G2 through G4 in Fig. 1A, bottom) in which the native binding pose was reached without any biasing force (Fig. 1F). As stated above, the G1 simulations began from the DFG-out/AL-open autoinhibitory conformation of the apo kinase (State 1; Fig. 1D), for which a representative crystal structure is available^17^, and each subsequent set of simulations was initiated from a snapshot generated from the preceding set. Based on the current understanding of imatinib binding, we believe these snapshots are representative of key intermediate states of binding (Fig. 1A)^3^.

In 15 of the 20 G1 simulations, an imatinib molecule entered the ATP-binding site of Abl and remained there for 1.5–37.5 µs, and in many such cases, imatinib remained in the ATP-binding site through the remainder of the simulation. Without exception, imatinib entry to the ATP-binding site was from the hinge side as opposed to the side of the αC helix. In two of the simulations (41 µs and 66 µs in length), an imatinib molecule entered the ATP-binding site. Although imatinib remained in the ATP-binding site to the ends of the simulations (for 37 µs and 61 µs, respectively) in a pose that runs approximately parallel to the native pose, this conformational state (which we sometimes refer to as State 2; Fig. 1D) is most likely transient, as it deviates substantially from the native imatinib binding pose. In these two simulations, the autoinhibitory conformation remained stable, with a stable DFG-out conformation and AL-open conformation characterized by the presence of the β9 strand C-terminal to the DFG motif. The imatinib pose has a root-mean-square deviation (RMSD) of ~8 Å (Fig. 1E) from the native pose, differing from the native pose in that it places the imatinib in a position that is more solvent-exposed and where the imatinib benzamide moiety is not hydrogen bonding with the hinge of Abl kinase.

Conjecturing that State 2 may represent an intermediate state in imatinib binding, we launched five further (G2) simulations from this pose. We deprotonated Asp381 in the G2 simulations, as Asp381 is solvent-exposed and likely deprotonated in the native imatinib-bound state^12,13^. (The estimated pKa of Asp381 in the imatinib-bound state was reported to be 4.4^3^.) We thus surmised that Asp381 deprotonation occurs as this residue becomes solvent exposed. Deprotonation likely destabilizes the autoinhibitory conformation and would help induce the transition to the imatinib-bound conformation in the simulations. In one of these simulations, the imatinib molecule adopted a more buried pose, with the hinge hydrogen bonds formed and the middle moiety of imatinib buried by the DFG motif (we sometimes refer to this conformation as State 3; Fig. 1D), and the RMSD of this pose to the native pose dropped to 4 Å or less (Fig. 1E). Recognizing the potential importance of this pose, we used it as a starting point for five further simulations (G3). In one of the G3 simulations, imatinib reached a native-like pose, with its piperazine moiety buried further behind the β9 strand, portending disruption of the AL-open conformation (we sometimes refer to this as State 4; Fig. 1D). This native-like pose and the native pose are highly similar, except for substantial differences in the conformation of the A-loop (Fig. 1G).

Unbound imatinib is not protonated at its piperazine moiety when in solvent^18^, but it is protonated when bound with Abl (Supplementary Fig. 2C)^3^. We thus launched an additional 10 simulations (G4) from the native-like pose (State 4), with the N34 atom of the piperazine moiety protonated; this moiety was located adjacent to Glu286 and Glu282 of the αC helix, and Asp381 of the DFG motif. In 8 of the 10 simulations, both the conformation of Abl and the binding pose of imatinib remained largely stable. In the other two G4 simulations, the A-loop closed, with the β9 strand at the N-terminal end of the A-loop being displaced by the piperazine moiety and rotating ~90° (Fig. 1G), and imatinib arriving at the native pose (we sometimes refer to this conformation as State 5; Fig. 1D and F). In these two simulations, in which imatinib disrupted the β6–β9 sheet, a hydrogen bond was formed between N34 and the β6 strand, thereby inducing the A-loop to close. In the open conformation of the A-loop, it thus appears that disrupting the β6-β9 sheet (β6 is N-terminal to the catalytic loop) is a critical energetic barrier to A-loop closing. Imatinib subsequently remained stable in the native conformation until the ends of these two simulations (~60 µs each).

The RMSD with respect to the Abl-imatinib crystal structure showed that the simulation-generated Abl-imatinib structure is highly consistent with the crystal structure, both in terms of the imatinib pose (<2 Å) and in terms of the A-loop conformation (~5 Å; Fig. 1E). As shown, the closing of the A-loop occurred after imatinib had largely settled in the native pose, consistent with the induced-fit nature of the conformational change being crucial for imatinib binding^5,15^. This induced-fit step involves closing of the A-loop, and is illustrated in Fig. 1A as the transition from the DFG-out/AL-open·I state (corresponding to the native-like pose) to the DFG-out/AL-closed·I state (the native pose) in the binding diagram.

An important assumption for our simulations of imatinib binding is that Asp381 protonation favors the DFG-out/AL-open conformation. To corroborate this assumption, we performed 25 simulations (lasting 417 µs in aggregate) starting from State 1 (i.e., from the DFG-out/AL-open conformation with Asp381 protonated) without imatinib present. As expected, the DFG-out/AL-open conformation remained stable in all these simulations. For comparison, we performed another 20 simulations, which were set up identically to the initial 25 except that Asp381 was deprotonated, and we found that a transition to the DFG-in/AL-open conformation occurred in two of these simulations. We did not observe closing of the A-loop in any of our simulations starting from the DFG-out/AL-open conformation, regardless of the protonation state of Asp381. Together, these simulations suggest that spontaneous closing of the A-loop is likely to be rare without imatinib binding, and that protonation of Asp 381 stabilizes the DFG-out conformation (Supplementary Fig. 2D).

These four generations of simulations together thus captured the entire process of Abl-imatinib binding. Concatenation of selected simulations, with one selected from each generation (the concatenated binding simulation), produced an aggregate simulation of imatinib binding in which imatinib first selectively recognizes the autoinhibitory conformation of Abl and settles in the ATP-binding site. Imatinib then induces closing of the A-loop, suggesting that Abl-imatinib binding is a hybrid process of conformational selection and induced fit.

**Fig. 3:**
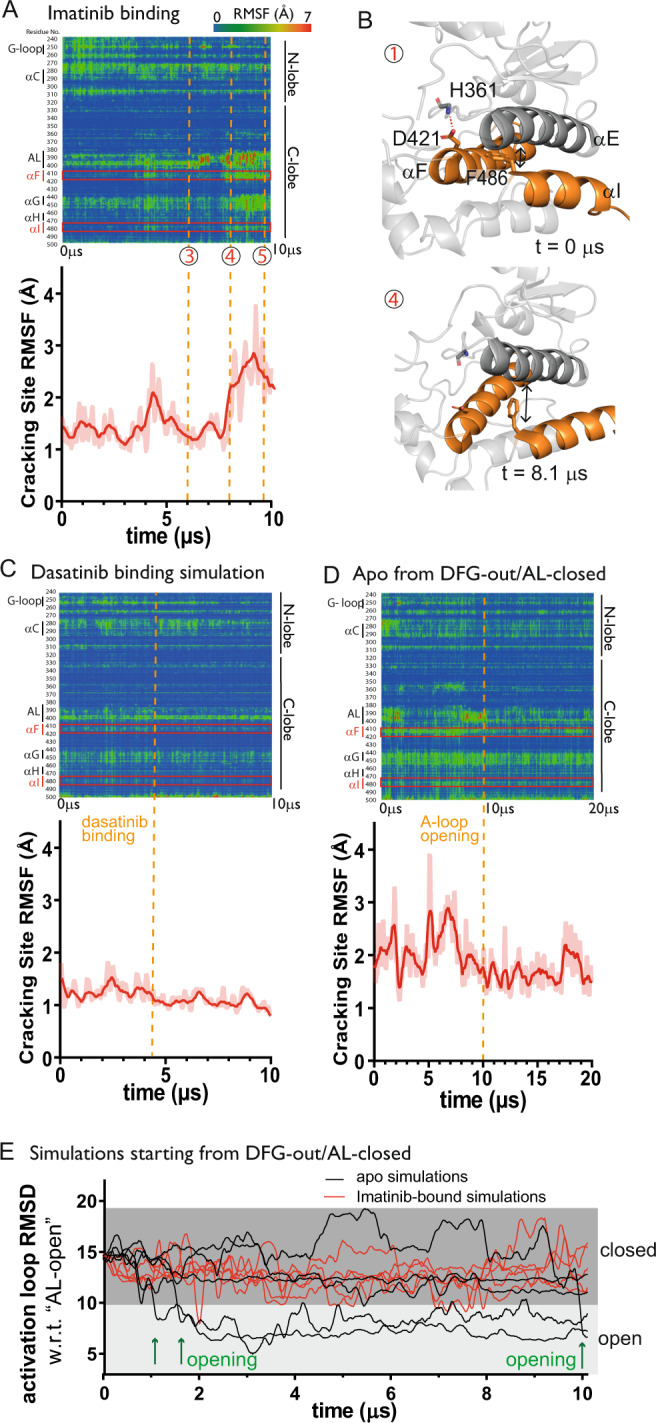
Cracking at the C-lobe in the Abl-imatinib binding simulation. **A** Top: Root mean square fluctuation (RMSF) of every residue as a function of time from the concatenated binding simulation (shown in Fig. 1A). The cracking site at the C-lobe is highlighted with the red rectangles. Bottom: Cracking site (residues 412–419, 477–483) RMSF over time. (RMSF values are shown as transparent lines for each frame; smoothing was applied and shown as a solid line to highlight the general trends in the data.) Time points 3–5 refer to the states marked in Fig. 1. **B** Snapshots of the cracking site in State 1 and State 4 in the concatenated binding simulation. **C** Similar analysis as shown in panel (A), but for a dasatinib-binding simulation. **D** Similar analysis as shown in **A**, but for a simulation of apo Abl kinase starting from the imatinib-bound crystal structure (i.e., with the imatinib removed). As shown, the cracking subsides after A-loop closing. **E** A-loop RMSD with respect to the active conformation (DFG-in/AL-open state; PDB ID: 2F4J) as a function of time in the simulations starting from the crystal structure of the Abl-imatinib complex (PDB ID: 1OPJ), with (red) or without (black) removing the imatinib. The events of A-loop opening in the apo simulations are marked with arrows.

Type II kinase inhibitors such as imatinib are known to disrupt the conserved regulatory spine of kinases, thereby severing links between the N-lobe and the C-lobe^19,20^. This disruption of the interactions between the two lobes is reflected in our simulations of imatinib binding, in which we observed the A-loop become wedged between these lobes during A-loop closing. (As shown in Supplementary Fig. 1B, this mode of binding is distinct from that of type I kinase inhibitors such as dasatinib.) The A-loop is disordered in some crystal structures of the Abl-imatinib complex, but in others it adopts a conformation with well-defined secondary structure. The B-factors of the resolved A-loops in the crystal structures tend to be among the highest in the kinase. In addition, NMR measurements of imatinib in complex with Src kinase, a close homolog of Abl kinase, failed to detect resonance from the A-loop, suggesting that this region adopts highly heterogeneous conformations when the kinase is bound to imatinib in solvent^21^. Furthermore, it is known that in the inactive, imatinib-bound conformation of Abl, the A-loop is not phosphorylated, and this allows it to assume different conformations^20,22^. In our simulations of Abl-imatinib binding, the A-loop—and especially its middle and C-terminal regions—remained highly flexible after binding (Fig. 3A). Further, in our simulations starting from an Abl-imatinib complex (PDB ID: 1OPJ), the A-loop was similarly flexible (Supplementary Fig. 2E), and its initial secondary structures quickly dissolved, despite the presence of bound imatinib. These simulation observations are consistent with the idea of conformational heterogeneity of the A-loop in the imatinib-bound state. We additionally simulated the same complex in the context of its crystal lattice and found that the A-loop remained highly stable (Supplementary Fig. 2F), supporting the notion that the simulations provide a reasonable description of the loop, and that crystal packing suppressed the A-loop flexibility that is present in solution.

### The DFG flip is central to the conformational selection step of Abl-imatinib binding

Having arrived at the native Abl-imatinib structure through a series of simulations (G1–G4), we next conducted a more detailed analysis of the 20 G0 simulations starting from the active, DFG-in/AL-open apo Abl kinase conformation (State 0). We observed that these simulations can also arrive at State 1 and 2 (Fig. 2A), and from there potentially arrive at the native Abl-imatinib structure. In 13 of the 20 simulations, Abl underwent the DFG flip and arrived at State 1 (Fig. 2A and Fig. 2C, left panel). The DFG flip appears to be independent of imatinib, as it occurred in the absence of imatinib in the binding site. In the other 7 G0 simulations, the DFG flip did not occur, presumably due to the stochastic nature of the DFG flip.

In two of the 13 G0 simulations in which a spontaneous DFG flip occurred (Fig. 2B), an imatinib molecule subsequently entered the ATP-binding site and arrived at a conformation highly consistent with State 2 (Fig. 2C, right panel), which had been independently produced by the G1 simulations (Fig. 1A). In the other 11 of these 13 simulations, imatinib did not adopt any stable and persistent pose, presumably because the simulations were relatively short compared to the timescale of the process. From the G0 simulations, it is clear that the conformational selection step of imatinib binding is associated with the DFG flip (Fig. 2A and 2B). Having simulated imatinib binding from the active conformation to State 2 (in the G0 simulations), and from State 2 to the native binding state (in the G2, G3, and G4 simulations combined), we conclude that our simulated complex can arrive at the native complex structure regardless of whether the simulations were initiated from the active conformation or from the autoinhibitory conformation of Abl.

### The C-lobe undergoes cracking as the A-loop closes with imatinib binding

The C-lobes of protein kinases are considered more stable than the N-lobes^23,24^. Previous MD simulations of the transition of EGFR kinase from its active conformation to its inactive one, however, have shown that the conformational change involves “cracking” of the C-lobe^25,26^. The term cracking was introduced to refer to local loss of native residue-residue interactions, compensated by increased local entropy, in intermediate states of a large conformational change in a protein. In C-lobe cracking, native residue-residue contacts of the C-lobe are substantially disrupted, giving rise to high local conformational fluctuations, which entropically compensate for the C-lobe instability^25^. Analysis of our Abl-imatinib binding simulations shows that similar C-lobe cracking occurred with imatinib binding after the imatinib arrived at the intermediate pose of binding (State 3), and especially just before and after closing of the A-loop (i.e., the transition from State 4 to State 5; Fig. 3A). Our analysis also shows that the cracking largely distorted the packing of the αE helix with the αF and αI helices (Fig. 3B and S4A). In addition, we observed cracking of the αG helix—a behavior that has been reported previously^5^. After the A-loop closed and imatinib arrived at the native pose, most of the native residue-residue contacts were recovered and local conformational fluctuations reduced (Supplementary Fig. 5A). Some of the native residue-residue contacts remained disrupted, however, indicating that the C-lobe retained some degree of instability in the imatinib-bound state (Fig. 3A).

We previously simulated the binding of another tyrosine kinase inhibitor, dasatinib, to Src kinase, and observed a dasatinib molecule settle in its native pose in the ATP-binding site^2^. For comparison with Abl-imatinib binding, we also simulated the binding of dasatinib with Abl kinase, starting from the active conformation. In 20 simulations (of ~107 µs total), one binding event was observed in which dasatinib arrived at the native binding pose and remained there to the end of the simulation (Supplementary Fig. 3A–B and Supplementary Movie 2). Unlike imatinib, dasatinib bound to the active conformation of Abl and the binding process did not involve a concomitant conformational change of Abl. In the dasatinib-bound structure, the A-loop of Abl is phosphorylated and stabilized in the open conformation, whereas imatinib binding is mutually exclusive with this phosphorylation (Supplementary Fig. 1B). In addition, dasatinib binding did not involve cracking; the C-lobe of Abl remained stable throughout the entirety of the simulation (Fig. 3C).

To further investigate the presence of C-lobe instability in the DFG-out/AL-closed conformation, we performed 10 simulations of 10 µs each, starting from an imatinib-bound conformation of Abl (PDB ID: 1OPJ), of which five were apo and five were imatinib-bound. In all 10 simulations, the C-lobe initially exhibited a degree of conformational fluctuation similar to what we had previously observed in the imatinib-bound conformation of our binding simulations. In the imatinib-bound simulations, this behavior continued throughout the simulations and the A-loop remained closed, but in three of the five apo simulations the A-loop switched to the open conformation (Fig. 3E), strongly suggesting that the DFG-out/AL-closed conformation is energetically unfavorable without imatinib bound (this may be due to the aforementioned structural instability that is associated with this conformation). Cracking was observed during A-loop opening, and after the A-loop settled into the open conformation the conformational fluctuations subsided noticeably (Fig. 3D). These simulations and analyses further suggest that the C-lobe of Abl kinase is unstable in the DFG-out/AL-closed conformation, and that this instability is energetically compensated by imatinib binding.

### Imatinib-resistance mutations at the cracking region destabilize the C-lobe

**Fig. 4:**
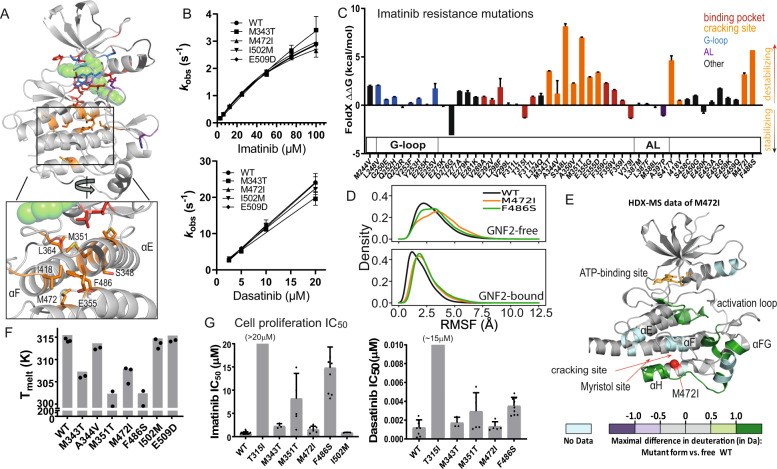
Mapping of imatinib-resistance mutations to the cracking site. **A** Top: Previously reported imatinib-resistance mutations mapped to an Abl-imatinib complex structure (PDB ID: 1OPJ). Bottom: A close-up view of the cracking site. The mutations are colored according to their positions in a manner consistent with **C**. **B** Observed binding rate constant (k_obs_) from stopped-flow experiments with increasing concentrations of imatinib (top panel, WT, *n* = 4; M343T, M472I, *n* = 3; I502M, E509D, *n* = 2 independent experiments) and dasatinib (bottom panel, *n* = 4 independent experiments). Data are presented as mean values ± SD (SD is shown only where the sample size is >3). Only the kinase domain of Abl was used. In vitro data of F486S is absent in this study because we failed to express and purify this mutant. Source data are provided as a Source Data file. **C** The FoldX estimates of the effects of the imatinib-resistance mutations on the folding free energy of Abl kinase (*n* = 3 independent estimations; data are presented as mean values ± SD). The values of mutations of different regions of Abl kinase are shown in different colors. Source data are provided as a Source Data file. **D** Normalized histograms of the RMSFs of the cracking region with respect to the averaged conformation of the region. The N-lobe and the αE and αF helices were aligned for the RMSD calculations. The top panel refers to simulations of Abl kinase bound with imatinib (PDB ID: 1OPJ), and the bottom panel refers to simulations of Abl kinase bound with both imatinib and GNF-2 (PDB ID: 3K5V). **E** HDX differences (D_M472I_ – D_wt_) between the WT and M472I mutant of Abl kinase, as measured by MS, mapped to the Abl kinase structure according to the color scheme shown (raw data is presented in Supplementary Fig. 4B). The alpha carbon of M472I is represented as a red sphere. **F** Melting temperatures of WT Abl kinase and seven imatinib resistance mutants (for WT, M372I, and I502M, *n* = 3; for all other constructs *n* = 2 independent experiments). Source data are provided as a Source Data file. **G** Cell proliferation IC_50_ values of imatinib (left panel: WT, *n* = 19; T315I, *n* = 12; M343T, *n* = 3; M351T, *n* = 6; M472I, *n* = 7; F486S, *n* = 11; I502M, *n* = 3 independent experiments) and dasatinib (right panel: WT, *n* = 8; T315I, *n* = 1; M343T, *n* = 3; M351T, *n* = 6; M472I, *n* = 5; F486S, *n* = 9 independent experiments) with cells expressing either WT Abl or the indicated imatinib-resistance mutants. Data are presented as mean values ± SD (SD is shown only when sample size is >3).

Imatinib induces complete remission in almost all patients in the chronic phase of CML who are treated immediately upon diagnosis^27^. When treated during the more aggressive stage of blast crisis, however, the disease in most patients ultimately becomes drug resistant^28,29^. The majority of relapsed patients harbor mutations within the Bcr-Abl kinase domain^8^. Many of these mutations are located at or near the ATP-binding site, of which the T315I (so-called “gatekeeper”) mutation is the most common, with 240 entries reported in the Catalogue of Somatic Mutations in Cancer (COSMIC, release v96)^30^. Structurally, it is not surprising that these mutations can disrupt imatinib binding, as they remove favorable interactions or introduce unfavorable interactions between the kinase and the drug. In addition to the mutations near the ATP-binding site, there is a group of distal imatinib-resistance mutations clustered in the C-lobe (Fig. 4A) at the αE, αF, αH, and αI helices (e.g., M343T/I, M351T, F359C, V379E/A, M472I). These mutations constitute a substantial subset of all imatinib-resistance mutations. Altogether, they account for 162 entries in the COSMIC database, with M351I being the fifth-most prevalent imatinib-resistance mutation overall (92 entries). Despite their prevalence, the mechanism by which these mutations confer imatinib resistance has remained unclear.

It has been previously demonstrated that imatinib binding to wild-type (WT) Abl occurs in a two-step process, as indicated by stoped-flow measurements of the kinetics of binding^3^. The observed binding on-rate (k_obs_) begins to deviate from linearity with increasing concentrations of imatinib, indicating a rate-limiting step associated with conformational change in the binding. In the present study, we found that imatinib binding to Abl with resistance mutations at the cracking site has similar binding kinetics at low imatinib concentrations (Fig. 4B, top panel), suggesting that the C-lobe mutations do not affect the first (conformational-selection) step of binding. The binding rates of dasatinib with the Abl mutants, on the other hand, show no deviation from linearity, even at high dasatinib concentrations (Fig. 4B, bottom panel). This is consistent with our previous finding that dasatinib binding does not involve large conformational changes of the protein.

It is notable that the imatinib resistance mutations in the C-lobe are located at the region of C-lobe cracking that occurs upon imatinib binding. They tend to be at the interior of the C-lobe and appear to weaken the hydrophobic packing (e.g., M343T and M351T). We used FoldX software^31^ to estimate the imatinib-resistance mutations’ effects on the thermodynamic stability of Abl kinase and found that the mutations mostly reduce its stability; further, the C-lobe imatinib-resistance mutations tend to destabilize the kinase more than other resistance mutations (Fig. 4C).

We then conducted simulations of WT, M472I, and F486S Abl kinase bound to imatinib, and observed that the two imatinib-resistance mutations resulted in more pronounced deviations from the native structure in the cracking region than in the WT (Fig. 4D, top panel). The destabilizing effect of these mutations may result in increased interaction with HSP90, an abundant molecular chaperone that stabilizes client proteins under various cellular stress conditions. HSP90 is known to help preserve structurally compromised protein kinases (including Abl), and to facilitate their conformational changes^32,33^. We also used hydrogen-deuterium exchange mass spectrometry (HDX-MS) to probe the effects of the M472I mutation (as an example of a C-lobe mutant) (Supplementary Table 1). The results confirmed that M472I increases HDX in the C-lobe of the kinase in the apo form (Fig. 4E), confirming a higher conformational instability for the mutant. Specifically, higher HDX of the M472I mutant was seen in part of the αH helix and the connected αG-αH linker (Leu452–Glu470), the αE helix and the catalytic loop (Tyr353–Leu370), and at the αEF helix C-terminal to the A-loop (Ile402–Ser410). The latter two regions are distal to M472I, suggesting a non-local effect of the mutation. Interestingly, these regions with increased HDX are spatially contiguous and connect the mutation site to the imatinib-binding site, hinting at an allosteric mechanism by which M472I hinders A-loop opening and imatinib binding (Supplementary Fig. 4C).

We also performed HDX-MS experiments on the A344V mutant (Supplementary Fig. 4A), and found that this mutation also destabilizes Abl, but to a much lesser degree than M472I. This result is consistent with FoldX estimates indicating that the effect of A344V on the folding energy of Abl kinase is about a third that of M472I (Fig. 4C). Previous analysis indicates that HDX in proteins can occur on the sub-100-ps timescale in localized conformational fluctuations^34^. We infer that the differential degree of conformational fluctuation we observed in our simulations may underlie the HDX-MS data, despite the timescale gap between the HDX-MD measurements (seconds) and the simulations.

Further, we tested whether each of four imatinib-resistance mutations at the cracking region (M343T, M351T, M472I, or F486S) could impact the effects of imatinib or dasatinib in cellular proliferation assays. We found that two of the cracking site mutations (M351T and F486S) conferred substantial imatinib resistance in cellular assays (Fig. 4G, left panel), and led to higher IC_50_ values in kinase inhibition assays compared to WT (Supplementary Fig. 4D). In addition, FoldX estimates indicate that these mutations destabilize the kinase more strongly than the other C-lobe imatinib-resistance mutations (Fig. 4C). The apparent correlation of the estimated destabilization effect with imatinib resistance supports the idea that maintaining stability of the C-lobe is crucial for imatinib binding to Abl. We also found that the cracking region mutations had relatively little effect on dasatinib activity (Fig. 4G, right panel). This is in contrast to the gatekeeper mutation (T315I) at the ATP-binding site, which confers resistance to both imatinib and dasatinib.

### Melting-temperature measurements confirm the destabilizing effect of the C-lobe mutations

We measured the effects of seven imatinib-resistance mutations on the melting temperature of Abl kinase (Fig. 4F). The results show that the imatinib-resistance mutations at the C-lobe cracking region (M343T, M351T, M472I, and F486S) reduced the melting temperature of the WT kinase (315 K) by 8–13 K, in agreement with the notion that the imatinib-resistance mutations weaken the stability of Abl kinase. Consistent with our FoldX estimates, A344V only reduced the melting temperature of Abl kinase by 2 K (Fig. 4F). In addition, M343T and M472I mutations, which impacted the imatinib IC_50_ value to a lesser degree than M351T and F486S (Fig. 4G left panel and Supplementary Fig. 4D), also had a lesser effect than M351T and F486S on protein stability in FoldX predictions and melting experiments (Fig. 4C and Fig. 4F). These results support the notion that the C-lobe mutations produce imatinib resistance by structurally destabilizing Abl kinase. As controls, we tested the effects of I502M and E509D, two additional imatinib resistance mutations not in the cracking region, and found that they did not affect the melting temperature of the kinase. These findings confirm that the resistance-conferring mutations at the cracking region tend to weaken the structural stability of Abl. Importantly, our simulations suggest that these mutations destabilize the DFG-out/AL-closed (imatinib-bound) state of Abl (Fig. 4D, upper panel). Although they can also destabilize the active state (and potentially the other states), this happens to a far lesser degree (Supplementary Fig. 5B). These mutations can thus hinder imatinib binding without disrupting kinase activity.

A number of small-molecule Abl inhibitors, such as GNF-2 and its close analog GNF-5^35^, bind at the myristoyl-binding site^10^, and we wanted to determine if their binding could allosterically affect imatinib binding. Additionally, we wanted to investigate whether small-molecule binding to the myrisitoyl-binding site affects the stability of the C-lobe in the imatinib-bound state. We thus simulated WT, M472I, and F486S Abl kinase bound to both imatinib and GNF-2 (GNF-2 binding to Abl is known to be mutually accommodating to either dasatinib or imatinib binding)^35^, and analyzed the allosteric coupling between the GNF-2-binding site and the imatinib-binding site using N-body Information Theory analysis^36^ (Supplementary Fig. 5C). We found indications of allosteric coupling between these two sites, and though the coupling was weak, it was above the level of noise. The allostery was unaffected by GNF-2 binding, or by M472I or F486S mutation, suggesting that the effect of the mutations on imatinib binding is not mediated by the interaction captured in this analysis.

**Fig. 5:**
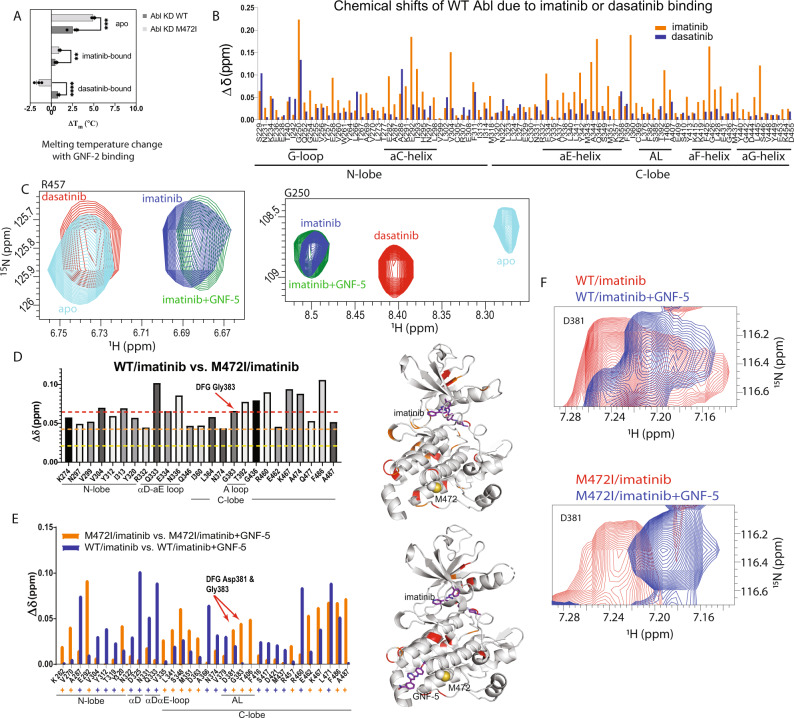
Melting-temperature and NMR analyses of the M472I mutation and the binding of imatinib, dasatinib, and GNF inhibitors. **A** Melting-temperature changes resulting from GNF-2 binding to apo, imatinib-bound, and dasatinib-bound WT Abl kinase and the M472I mutant (*n* = 3 independent experiments; ** indicates *P* ≤ 0.01, *** indicates *P* ≤ 0.001, and **** indicates *P* ≤ 0.0001; two-tailed *t*-test with 95% confidence interval was performed; apo: *P* = 0.001, degrees of freedom (df) = 5.52, effect size (d) = 5.9; imatinib bound: *P* = 0.003, df = 8.2, *d* = 4.1; dasatinib bound: *P* = 0.00004, df = 6.62, *d* = 9.5). Data are presented as mean values ± SEM. Source data are provided as a Source Data file. **B** Chemical shifts of WT Abl kinase due to imatinib or dasatinib binding. **C** NMR spectra of R457 and G250 in apo, imatinib-bound, dasatinib-bound, and imatinib+GNF-5-bound Abl kinase. **D** Comparison of imatinib-bound WT and M472I Abl kinase. The residues that underwent large chemical shifts due to M472I mutation are shown in the right panel in a ribbon diagram. The yellow, orange, and red dashed lines mark shifts of more than one, two, and three standard deviations, respectively. The residues that displayed chemical shifts above the red or the orange threshold are shown in the right panel in the same color. **E** Chemical shifts associated with GNF-5 binding to WT and M472I Abl kinase. The residues for which WT and M472I behave differently on GNF-5 binding (with differences in chemical shifts of more than one standard deviation) are shown in the image to the right. **F** NMR spectra of D381 of the DFG motif in imatinib-bound and imatinib+GNF-5-bound WT (upper) and M472I (lower) Abl kinase.

The simulations also showed that GNF-2 stabilized the cracking region, and compensated for the destabilizing effect of the two mutations (Fig. 4D, bottom panel). This is unsurprising, as the myristoyl-binding site occupied by GNF-2 and GNF-5 is located near the cracking region. We measured the effect of GNF-2 binding on the melting temperature of Abl kinase to investigate the predicted structural instability associated with the imatinib-resistance mutations at the C-lobe. We reasoned that the stabilizing effect of GNF-2 should be positively correlated to instability at the C-lobe. We observed a greater increase in melting temperature associated with GNF-2 binding to M472I than to the WT kinase (Fig. 5A), which is consistent with the predicted destabilization effect of M472I. Similarly, the melting temperature increase from GNF-2 binding to imatinib-bound Abl is also larger for M472I than for WT. In contrast, the melting temperature increase from GNF-2 binding to dasatinib-bound Abl is also smaller for M472I than for WT (Fig. 5A). These findings together are consistent with the notion that M472I affects imatinib binding by destabilizing the C-lobe, and bound imatinib, as opposed to bound dasatinib, also destabilizes the C-lobe.

### NMR analyses corroborated the effect of imatinib binding on C-lobe packing

To gain further insight into the effects of imatinib binding on the stability of the C-lobe, we used NMR analysis to investigate WT Abl kinase alone or in the presence of imatinib or dasatinib. Imatinib binding changed the chemical shifts of WT Abl kinase more globally and to a greater degree than dasatinib binding (Fig. 5B), which is not surprising given the large conformational change of the A-loop that occurs with imatinib binding. Conformational change was not limited to the A-loop, however, as we also observed significant chemical-shift perturbations throughout the N-lobe and C-lobe (Fig. 5B). The observed chemical-shift perturbation of R457 (Fig. 5C), for example, reflects a change in the chemical environment of the residue, possibly due to conformational changes in nearby regions (including helices αE and αF) that are involved in the structural destabilization of the C-lobe; these regions are distal to both the imatinib-binding site and the A-loop. (The chemical-shift perturbations observed in the αE and αF helices (Fig. 5B) are consistent with the notion that imatinib binding affects helical packing at the C-lobe.) We also observed a significant chemical-shift perturbation for G250 in the glycine-rich loop (G-loop), a sub-region of the ATP-binding site that interacts with the ATP phosphate groups. The NMR data suggest that binding of dasatinib and imatinib have distinct effects on the conformation of the G-loop (Figs. 5B and 5C).

We next investigated the effect of M472I mutation on imatinib binding. NMR analysis (Fig. 5D) showed that M472I led to chemical-shift perturbations of many residues of the C-lobe cracking region that are not adjacent to the mutation site, consistent with the notion that M472I destabilizes the C-lobe. Moreover, the conformational effects of M472I extend beyond the C-lobe (Fig. 5D), even reaching the N-lobe β strands. The unexpected chemical-shift perturbation of Gly383 in the DFG motif of this mutant (Fig. 5D) is consistent with the hypothesis that imatinib resistance arising from C-lobe mutations is rooted in hindered conformational changes of the DFG motif and the A-loop.

The melting-temperature experiments showed that the effect of GNF-2 binding is greater on the M472I mutant than on WT Abl kinase (Fig. 5A). This was also observed in the NMR analysis (Fig. 5E) of GNF-5 binding (which is identical to GNF-2 in the moiety in contact with Abl). GNF-5 binding generally produced larger chemical-shift perturbations at the C-lobe of imatinib-bound M472I than in imatinib-bound WT. Importantly, GNF-5 appears to produce larger chemical-shift perturbations at D381 of the DFG motif in M472I Abl kinase than in the WT (Fig. 5F). These findings are consistent with the notion that binding of GNF inhibitors is expected to stabilize the C-lobe, and is thus more impactful on the M472I mutant because this mutation destabilizes C-lobe packing. Taken together, the melting-temperature and NMR analyses provide strong support for our hypothesis, based on MD simulations, that imatinib binding destabilizes the C-lobe and that the C-lobe imatinib-resistance mutations hinder imatinib binding (and the associated conformational change) by weakening C-lobe packing.

## Discussion

In this study, we conducted simulations of imatinib and Abl kinase, starting from an unbound state and without using any biasing force, and arrived at a bound structure that is highly consistent with crystal structures of the Abl-imatinib complex. The simulations describe an atomic-level pathway of imatinib binding in which imatinib first selectively recognizes the autoinhibitory conformation of Abl kinase and enters the ATP-binding site. The protein then undergoes a large conformational change in which the A-loop closes, after which the system settles into the native Abl-imatinib structure. Given that this closing of the A-loop coincides with imatinib arriving at the native pose, and that the DFG-out/AL-closed conformation has not been observed in crystal structures of Abl without imatinib or an imatinib analog bound, we believe the conformational change represents an induced-fit step. (We note that the DFG-out/AL-closed conformation has been observed in structures of other kinases that are not bound with an inhibitor (e.g., IRK^37^), so we cannot exclude the possibility that, for Abl kinase, this is a rare conformation that is stabilized by imatinib.) This pathway of imatinib binding thus appears to involve both conformational selection and induced fit—a hybrid model that is consistent with findings from previous studies^3,5,15^—and our simulations provide new structural information about the intermediate states of imatinib binding.

With steady improvement in MD force fields and rapid growth in computational power, it is increasingly feasible to simulate protein–small-molecule binding using MD simulations. It has remained a challenge, however, for MD simulations to capture binding events that occur with substantial conformational change of the protein. Producing a concatenated simulation that recapitulates Abl-imatinib binding (a process that involves a large associated conformational change of the A-loop) provides a proof of principle that this challenge can be overcome.

Abl-imatinib binding has been previously studied using simulations^4,5^. These simulations started from the imatinib-bound structure, and used various biasing forces to promote unbinding and binding. In this study, we started our simulations from the unbound state, and arrived at the bound structure without using any biasing forces. The timescale of the simulated binding appears to be broadly consistent with Abl-imatinib binding kinetics. Our work differs from the previous studies in that we determined key intermediate states, or “milestones,” in the binding process from which additional simulations were launched to advance the process—an approach that resembles adaptive sampling methods^1,38,39^. Adaptive sampling methods, however, use an algorithmic approach to identify key intermediate states, whereas we used domain-specific knowledge, visual inspection of the simulations, and trial-and-error. At present, automated methods for identifying key intermediate states yield results of varying accuracy in the case of large conformational changes^39^, but further development of these methods could help obviate the need for laborious trial-and-error approaches in the future.

In the binding path from our simulations, imatinib enters the binding site from the hinge side of Abl kinase rather than from the C-helix side, as previously suggested^4^. We also observed that the closing of the A-loop that occurs with imatinib binding is accompanied by cracking in the C-lobe, and some degree of local structural instability remained in the imatinib-bound state. A number of mutations that are known to confer resistance to imatinib are clustered within a region of the C-lobe, but the mechanism by which these mutations confer resistance has been unknown. Based on our analyses, we propose from the linkage between the A-loop conformational change and C-lobe instability that these mutations confer imatinib resistance by reducing C-lobe stability and disfavoring the closed conformation of the A-loop that is associated with imatinib binding. The destabilizing effect may result from the disruption of the two conserved hydrophobic spines^19^ that connect the N-lobe and C-lobe, and anchor catalytically important regions of the kinase to a conserved and highly hydrophobic αF helix^40^. The imatinib-resistance mutations in the C-lobe are either part of or adjacent to this hydrophobic architecture, and our results suggest that these mutations affect imatinib binding by way of this network. This notion is consistent with the recent finding that many imatinib-resistance mutations increase Abl kinase dynamics and reduce the residence time of imatinib binding^41^. From a therapeutic perspective, our findings suggest that combining imatinib with small molecules that bind at the myristoyl-binding site (such as GNF-5) may counter the destabilizing effects of imatinib-resistance mutations at the C-lobe, and potentially overcome imatinib resistance caused by these mutations.

## Methods

### Molecular dynamics simulations

#### Generation 0 of Abl-imatinib binding simulations

The systems for the G0 simulations were prepared based on a crystal structure of Abl kinase in which the DFG motif is in the active “DFG-in” state and the A-loop is in the open state (i.e., DFG-in/AL-open; PDB ID: 2F4J)^42^. The DFG Asp residue was protonated and three imatinib molecules were placed at random locations not in contact with the kinase. The system was solvated in a cubic simulation box with periodic boundary conditions (~78.6 Å per side, containing ~60,000 atoms total). Water molecules were represented explicitly and Na^+^ and Cl^–^ ions were added to neutralize the system and achieve physiological levels of 150 mM each. The systems were parametrized with the a99SB-disp force field^43^, imatinib was parametrized using the general Amber force field^44^, and the systems were equilibrated on Desmond^45^ using a mixed NVT/NPT schedule. MD simulations were performed on the special purpose machine Anton 2^46^ in the NVT ensemble with *T* = 310 K using the Nosé–Hoover thermostat^47,48^. The simulation time step was 2.5 fs, and the r-RESPA integration method^49^ was used, with long-range electrostatics evaluated every three time steps. Electrostatic forces were calculated using the *u*-series method^50^ with a 1.37-nm cutoff for the electrostatic pairwise summation; a 0.9-nm cutoff was used for the van der Waals interactions.

#### Generation 1 of Abl-imatinib binding simulations

The system for the G1 simulations was prepared based on a crystal structure of apo Abl kinase in which the DFG motif is in the autoinhibitory state and the A-loop is in the open state (i.e., DFG-out/AL-open; PDB ID: 1OPK)^17^. The DFG Asp residue was protonated and three imatinib molecules were placed at random locations. The systems were solvated in a cubic simulation box with periodic boundary conditions (~86.4 Å per side, containing ~80,000 atoms total). The simulations were prepared, equilibrated, and run following the same methods used for the G0 simulations. Twenty independent unbiased simulations were conducted. In 11 of the 20 simulations, imatinib found the binding pocket (Fig. 1A).

#### Generations 2 and 3 of Abl-imatinib binding simulations

We then randomly selected a G1 simulation snapshot in which imatinib was in the binding pocket and in the correct orientation. At this point, we deprotonated the DFG Asp, which was preventing imatinib from becoming fully embedded in the binding pocket. Five independent simulations (G2) were extended from this pose with a similar system setup and protocol. Another five independent G3 simulations were extended from the last frame of one of the G2 simulations, with no changes to the system. (In the selected frame, which we conjectured to represent an intermediate conformation in imatinib binding, imatinib is in a pose highly similar to the native pose, but the A-loop is still in the open conformation).

#### Generation 4 of Abl-imatinib binding simulations

Once imatinib was fully embedded in the binding pocket, we observed that N34 of imatinib (the methylation site of the N-methylpiperazine group) was in a solvent-exposed environment, in close proximity to Glu286, that might allow for protonation of N34. We thus protonated N34 and extended five independent simulations (G4) from this point, following the same methods used for the G0 simulations.

#### Simulations starting from the DFG-out/AL-open conformation

The system was prepared based on the same crystal structure used for the G1 simulations (i.e., DFG-out/AL-open; PDB ID: 1OPK)^17^. DFG residue Asp381 was protonated. The systems were solvated in a cubic simulation box with periodic boundary conditions (~78.6 Å per side, containing ~60,000 atoms total). The simulations were prepared, equilibrated, and performed following the same methods used for the G0 simulations. 25 independent unbiased simulations were conducted, and the A-loop remained open throughout all of the simulations.

#### Abl-dasatinib binding simulations

The system for the dasatinib-Abl binding simulations were prepared based on the crystal structure of Abl kinase bound with dasatinib (PDB ID: 2GQG)^51^. Dasatinib was removed from the binding pocket and three dasatinib molecules were placed at random locations. The system was solvated in a cubic simulation box with periodic boundary conditions (~74.5 Å per side, containing ~55,000 atoms total). MD simulations were performed following the same methods used for the G0 simulations. Twenty independent 10-µs simulations were run.

#### Simulations starting from the DFG-out/AL-closed conformation

The systems for the simulations starting from the DFG-out/AL-closed conformation were prepared based on the crystal structure of Abl kinase bound with imatinib, in which the DFG motif is in the autoinhibitory state and the A-loop is closed (i.e., DFG-out/AL-closed; PDB ID: 1OPJ)^17^. Four systems were prepared based on the imatinib-bound WT Abl structure (PDB ID: 1OPJ): (1) WT bound with imatinib; (2) and (3) M472I and F486S mutants bound with imatinib; and (4) apo WT in the DFG-out/AL-closed conformation. The systems were solvated in cubic simulation boxes with periodic boundary conditions (~74.3 Å per side, containing ~50,000 atoms total). Five independent 20-µs simulations were run for each WT system and 10 independent 10-µs simulations were run for each mutant system. An additional five independent simulations of 10 µs each were performed with the system in which WT Abl kinase was bound with imatinib. We also simulated WT and the M472I and F486S mutants of Abl each simultaneously bound with imatinib and GNF-2 in the crystal pose from PDB ID: 3K5V^35^. The prepared systems contained ~70,000 atoms with a cubic simulation box of ~82 Å per side, and 10 independent 10-µs simulations were run for each construct. The simulations were performed following the same methods used for the G0 simulations.

#### Simulations starting from the DFG-in/AL-open conformation

The systems for the simulations starting from the DFG-in/AL-open conformation were prepared based on the crystal structure of the active conformation of Abl kinase (PDB ID: 2F4J)^42^. Three systems were prepared: (1) WT, (2) M472I, and (3) F486S Abl kinase. The systems were solvated in cubic simulation boxes with periodic boundary conditions (~80 Å per side, containing ~65,000 atoms total). Three independent 20-µs simulations were run for each system. The simulations were performed following the same methods used for the G0 simulations.

#### Crystal lattice simulations

We used the crystal structure of Abl kinase in complex with imatinib (PDB ID: 1IEP)^12^ to construct the crystal lattice for our MD simulations. The protein was crystallized in an F 222 space group with αβγ angles of 90°, which allowed us to satisfy the 90° angle requirements of the MD simulation box. We generated the crystal symmetry pairs using PyMOL Molecular Graphics System (Schrödinger, LLC) to build the full unit cell (containing 32 copies of the kinase). The system was solvated in simulation boxes with dimensions *a* = 112.89 Å, *b* = 147.37 Å, and *c* = 153.44 Å, and with periodic boundary conditions to match the unit cell dimensions with varying distance between the protein system and the first shell of the water molecules. Water molecules were represented explicitly and Na^+^ and Cl^–^ ions were added to neutralize the system and achieve concentrations of 150 mM each. The system, containing ~320,000 atoms, was equilibrated on Desmond^45^ using a mixed NVT/NPT schedule. Next, the system was simulated for 100 ns on Anton 2^46^ in the NPT ensemble with *T* = 277 K and a 2-fs step size (the temperature matching the reported crystallization temperature). The cell dimensions were monitored throughout the simulations and a system that sustained the reported crystal dimensions was picked to progress to the 10-μs production run, which was performed in the NVT ensemble (still at 277 K and with a 2-fs step size).

The simulation trajectories were visualized and analyzed using Visual Molecular Dynamics (VMD) software^52^, and the images of protein structures were made using the PyMOL Molecular Graphics System (Schrödinger, LLC).

### FoldX Calculations

FoldX is a computational tool used to predict the effects of mutations on protein stability, folding, and protein dynamics^31^. FoldX 4.0 was used to predict the effect of mutations on protein stability through the approximation of Gibbs free energy (ΔG). FoldX uses an empirical force field that is trained directly on experimental data. Prior to calculating free energies, we used the FoldX 4.0 Repair PDB command to optimize side-chain orientations for WT Abl kinase (PDB ID: 2F4J)^42^ atom coordinates^53^. The resulting coordinates were then processed to calculate the change in folding free energy of the mutants relative to the WT protein (ΔΔG_FoldX_ = ΔG_mut_ – ΔG_WT_). A negative ΔΔG_FoldX_ (ΔΔG_FoldX_ <0 kcal/mol) suggests that the mutant protein is more stable than the WT protein.

### Protein expression and purification

The kinase domain of human c-Abl (UniProt ID: P00519, residues 248–531), with human Abl 1a residue numbering^54^ was cloned into pET28 and modified to yield an N-terminal, TEV-cleavable His-tag^55^. The kinase domain mutations were introduced into the human c-Abl kinase domain by site-directed mutagenesis using the QuikChange II kit (Agilent, 200524) and verified by DNA sequencing.

Kinases were coexpressed in *Escherichia coli* BL21DE3 cells with full-length YopH phosphatase from *Yersinia pseudotuberculosis*^56^ and GroEL/Trigger factor, as previously described^55,57^. *E. coli* cells were grown in 2xYT media (Difco, BD 244020) at 37 °C for 4 h to a cell density of OD_600_ = 0.6. The temperature was subsequently reduced to 16 °C before cells were induced with IPTG (GoldBio, 12481C50) and grown overnight. Kinases were purified using Ni^+2^-affinity chromatography (HisTrap FF, GE Healthcare Life Sciences), anion-exchange chromatography (HiTrap Q FF, GE Healthcare Life Sciences), and size-exclusion chromatography (S75, GE Healthcare Life Sciences). Proteins in 20 mM Tris (pH 8.0; ChemCruz, sc-3715B), 250 mM NaCl, 5% glycerol, and 1 mM DTT were concentrated, frozen in liquid nitrogen, and stored at −80 °C. Protein purity was at least 95%, as determined by SDS-PAGE and Coomassie Blue staining.

### Thermal denaturation assays

Denaturation of WT Abl kinase and its mutants was monitored by changes in protein fluorescence using a Jobin Yvon Fluoromax-4 (Horiba) spectrofluorimeter exciting at 290 nm (bandpass, 2 nm) and scanning emission at 320–400 nm (bandpass, 8 nm), with an integration time of 0.1 s. Each protein was individually added to a final concentration of 1 μM in 1 mL of buffer containing 20 mM Tris (pH 8.0), 250 mM NaCl (Fisher, BP358-10), 5% glycerol, and 1 mM DTT. The samples were incubated for 1 min at each 1 °C from 10–80 °C before the fluorescence at each temperature was recorded. Protein fluorescence as a function of temperature was fit to a Boltzmann sigmoidal equation to obtain V50, or melting temperature.

### Biochemical IC_50_ measurements

Kinase activity was monitored using a continuous spectrophotometric assay, as described previously^58^. In brief, the consumption of ATP is coupled by way of the pyruvate kinase/lactate dehydrogenase enzyme reactions to the oxidation of NADH, which can be monitored through the decrease in absorption at 340 nm. Reactions contained 100 mM Tris (pH 8.0), 10 mM MgCl_2_ (Sigma-Aldrich, M9272) 0.5 mM ATP (Sigma-Aldrich, A2383) 1 mM phosphoenolpyruvate, 0.21 mg/mL NADH (Sigma-Aldrich, N8129), 75 U/mL pyruvate kinase, 105 U/mL lactate dehydrogenase, and 0.5 mM substrate peptide (sequence: AEEEIYGEFAKKK; RS Synthesis)^59^. Reactions were initiated through the addition of kinase at a final concentration of 33 nM, and the decrease in absorbance was monitored over 10 min at 30 °C in a spectrophotometer (SpectraMax 340PC384 and BioTek Neo2). The background activities of the proteins at different drug concentrations were determined in experiments without the substrate peptide, and subtracted from the kinase activities with the substrate peptide. To measure the IC_50_ for imatinib, imatinib (Ark Pharma, AK44930) was serially diluted threefold in 100% DMSO and added to the reactions to produce an 8-point dose-response curve ranging from 0–1 μM. The activity measured by the slope of NADH oxidation (mOD/min) was plotted verses the logarithm of the concentration of drug and fit to a sigmoidal equation with the hill slope set to 1.0.

### BCR-ABL mutagenesis and cell line generation

Full-length p210 *BCR-ABL* cDNA was sub-cloned into pEYK3.1, a retroviral vector, to construct the pEYKBA plasmid^60^. The pEYKBA plasmid was mutagenized by site-directed mutagenesis using the QuikChange II kit (Agilent) to create point mutations in the kinase domain of *BCR-ABL*. The pEYKBA plasmids were transfected into 293 T cells (ATCC, CRL-3216) using FuGENE HD (Promega). At 24 h after transfection retroviral supernatants were recovered as previously described^60,61^. The interleukin 3 (IL-3)-dependent hematopoietic pro-B cell line Ba/F3^62^ was transduced with the retroviral supernatant supplemented with 8 μg/mL of polybrene (Sigma) and IL-3-enriched media. Ba/F3 cells were deprived of IL-3 at 2 days post-infection to select for cells that had taken up the pEYKBA retrovirus. The Ba/F3 cells were a kind gift from Mohammad Azam (Cincinnati Children’s Hospital). They are commercially available at DSMZ (Leibniz Institute, DSMZ-German Collection of Microorganisms and Cell Cultures GmbH).

### Cell proliferation assays to determine IC_50_ values

0.5 × 10^4^ Ba/F3 cells expressing mutant Bcr-Abl proteins were plated in each well of 96-well plates in RPMI medium containing 10% FBS and lacking IL-3 (total volume, 100 μL). Imatinib (Ark Pharm) was included in the media in increasing concentrations (final concentrations ranging from 0–20 μM). Dasatinib (Cayman Chemical Company, 11498) was included in the media in increasing concentrations (final concentrations ranging from 0–10 μM). Viable cell numbers were assessed after 48 h using the WST-1 reagent (Roche), according to manufacturer’s specifications. Assays were performed in quadruplicate. A_450_ readings were plotted versus the logarithm of the concentration of drug and fit to a sigmoidal equation with the hill slope set to 1.0. The concentration of drug resulting in 50% maximal inhibition is reported as cellular IC_50_.

### Hydrogen-deuterium exchange mass spectrometry

Hydrogen exchange was performed as previously described^23^. A 50 pmol/µL stock solution of Abl kinase (WT, A344V, or M472I) was prepared in 20 mM Tris (pH 8.0), 150 mM NaCl, 1 mM DTT, and 5% glycerol in H_2_O. Deuterium exchange was initiated by dilution of each protein with 15-fold D_2_O buffer (pD 8.0) at room temperature. At each deuterium exchange time point, from 10 s to 4 h, an aliquot from the exchange reaction was removed and labeling was quenched by adjusting the pH to 2.5 with an equal volume of quench buffer (150 mM potassium phosphate in H_2_O). Quenched samples were immediately injected into the LC/MS system for mass analysis.

Each sample was analyzed as previously described^23,63^. Briefly, the samples were digested online using a Poroszyme immobilized pepsin cartridge (2.1 mm × 30 mm, Applied Biosystems) at 15 °C for 30 s, then injected into a custom Waters nanoACQUITY UPLC HDX ManagerTM and analyzed on a XEVO G2 mass spectrometer (Waters Corp., USA). The average amount of back-exchange using this experimental setup was 20%–30%, based on analysis of highly deuterated peptide standards. Deuterium levels were not corrected for back-exchange and are thus reported as relative^64^. All experiments were performed in duplicate. The error of measuring the mass of each peptide averaged ±0.12 Da in this experimental setup. The HDX-MS data were processed using PLGS 3.0 and DynamX 3.0 (Waters Corp., USA). Additional experimental details are provided in Supplementary Table 1 per the recommended format^65^. Detailed protocols for sample processing and data processing are provided in the Supplementary Methods.

### NMR measurements

#### WT Abl and M472I Abl protein sample preparation

Abl kinase (residues 251–533 of human c-Abl) was cloned, expressed, and purified in bacteria with additional co-expression of the GroEL chaperone and YopH phosphatase^55^. Briefly, Abl kinase containing a TEV-cleavable N-terminal His_6_-tag was co-transformed into *E. coli* BL21 (DE3) cells with plasmids expressing YopH phosphatase, GroEL, and Trigger factor. ^15^N Abl kinase samples for NMR experiments were produced in 100% ^2^H_2_O (Cambridge Isotope Labs, DLM-4-99-1000) M9 minimal media containing 1 g/L of ^15^NH_4_Cl (Cambridge Isotope Labs, NLM-467-50) and D-glucose as the sole nitrogen and carbon sources, respectively. The cells were grown at 37 °C to O.D_600_ ~0.6–0.8, cooled to 16 °C, treated with 1 mM IPTG to induce expression, and allowed to continue growing overnight.

Proteins were purified by Ni-NTA affinity chromatography followed by anion-exchange chromatography. The purified sample was concentrated by ultrafiltration and buffer exchanged into 50 mM Na/K pH 6.5, 50 mM NaCl, and 5 mM DTT. 10% ^2^H_2_O was added to the final NMR samples for a lock signal. ATP-competitive ligands (dasatinib and imatinib) and the myristoyl-binding-site ligand (GNF-5; Selleckchem, S7526) were added in excess to saturate the binding sites and stabilize particular Abl kinase states. DMSO was maintained at 5% for NMR samples containing ligands solubilized in DMSO.

#### Backbone relaxation experiments

All backbone (^1^H)-^15^N heteronuclear NMR experiments were acquired at 25 °C on a Bruker Avance III HD spectrometer operating at a ^1^H frequency of 850 MHz and equipped with a Triple Resonance (TCI) ^13^C-enhanced 5-mm cryogenic probe. Abl inhibitor (dasatinib, imatinib, or imatinib+GNF-5) ^1^H-^15^N samples were prepared at concentrations of 200–350 µM. Standard Bruker pulse sequence for ^1^H-^15^N TROSY-HSQC, *trosyf3gpphsi19.2*, was used^66–71^. All spectra were acquired with the following parameters: Scans = 64, Size of FID (F2:1 H/F1:15 N) = 2048/128, Spectral Width (F2:1 H/F1:15 N) = 15.9791 ppm/ 36 ppm. Sample stability prior and post backbone dynamics were assessed by acquiring 1D ^1^H spectra. The backbone assignments were downloaded from BMRB and transferred onto the Abl kinase WT with imatinib spectrum for the further analysis^72^. The chemical-shift differences were analyzed with the software CCPNMR, and graphed with Prism GraphPad 8.0.2.

### Stopped-flow kinetics experiments

We monitored inhibitor binding of imatinib and dasatinib by changes in protein fluorescence using a spectrofluorimeteric ligand-binding assay, as described previously^14^. Protein and buffer containing imatinib or dasatinib at varying concentrations was mixed at a 1:1 ratio in a total volume of 200 μL with at least a ten-fold molar excess of drug to obtain a pseudo first-order condition. Changes in protein fluorescence were monitored over time using a stopped-flow system (Applied Photophysics SX20). Protein fluorescence decay upon drug binding was fit to a single exponential equation to obtain the observed rate constant (*k*_obs_)^3^. The *k*_obs_ was plotted against drug concentration, and the association and dissociation rates were calculated based on the relationship: *k*_obs_ = *k*_on_[drug] + *k*_off_. A range of imatinib concentrations was used from 6 μM to 200 μM. The assay was limited to a maximal imatinib concentration of 200 μM due to imatinib solubility.

### Reporting summary

Further information on research design is available in the Nature Portfolio Reporting Summary linked to this article.

## Supplementary information

Supplementary Information file

Description to Additional Supplementary Information

Supplementary Movie 1

Supplementary Movie 2

Reporting summary

## Data Availability

Due to the large size of the molecular dynamics trajectories described in this work, they are available for non-commercial use through contacting trajectories@deshawresearch.com. The raw HDX-MS data generated in this study have been deposited in the ProteomeXchange Consortium via the PRIDE partner repository with the dataset identifier PXD034008. The PDB data used in this study are available under the following IDs: 1OPJ, 1OPK, 2F4J, 3K5V, 2GQG, and 1IEP. Source data are provided with this paper.

## Source data

Source data file
