# Supplementary material for: Structural mechanism of a drug-binding process involving a large conformational change of the protein target
Source: Nat Commun. 2023 Apr 5;14:1885. doi: 10.1038/s41467-023-36956-5 (PMC10076256; doi:10.1038/s41467-023-36956-5)
Supplement: Supplementary file 1 — Supplementary Information file [file 41467_2023_36956_MOESM1_ESM.pdf]

Supplementary Information

Structural mechanism of a drug-binding process involving a large conformational change of the protein target

Supplementary Figures

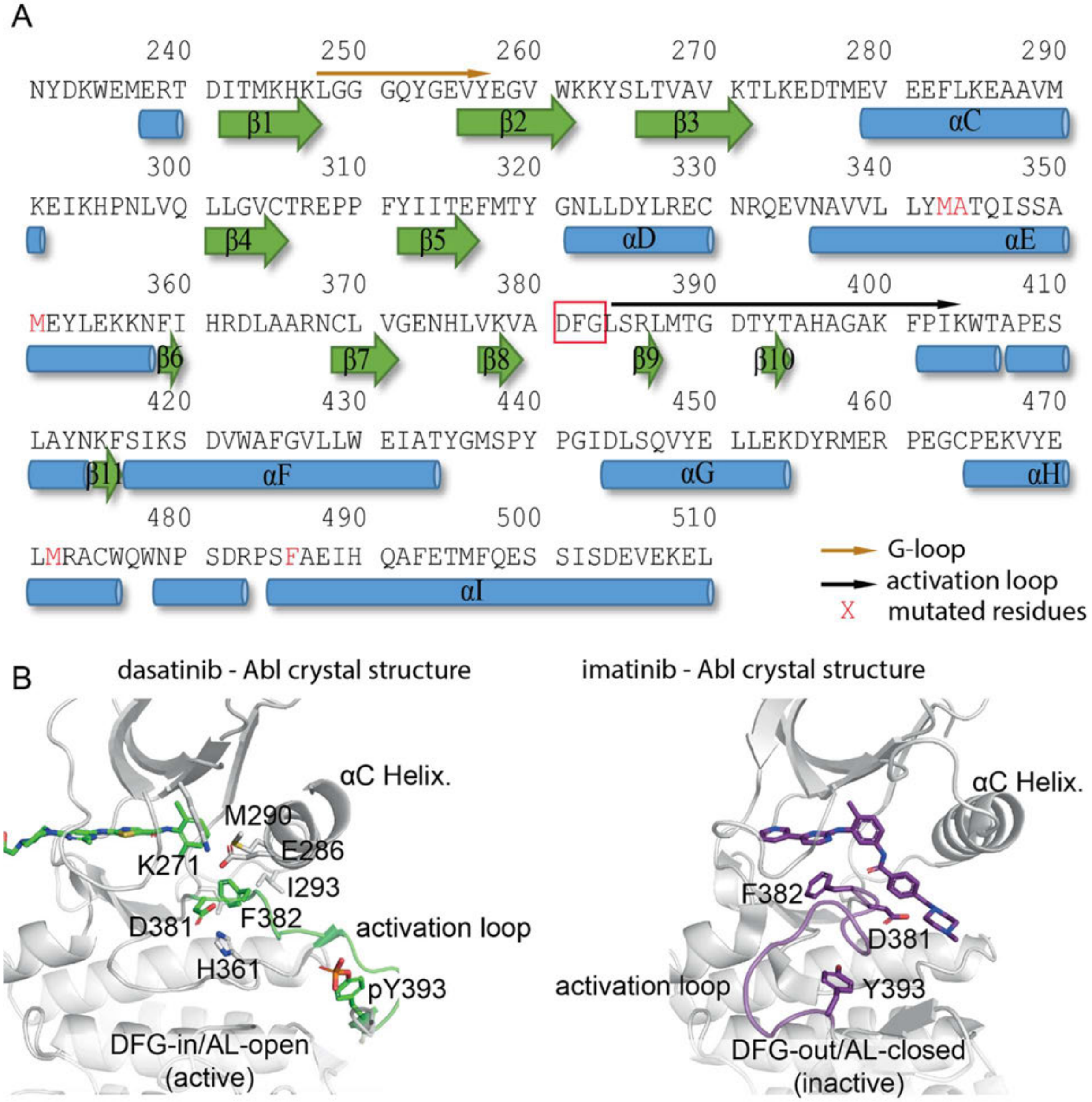

Supplementary Figure 1. (A) Sequence and the secondary structure view of the Abl kinase domain. Residues that were mutated in this study appear in red. The DFG motif is enclosed in a red rectangle. (B) Structural comparison, based on crystal structures, of Abl kinase bound with either imatinib (right, PDB ID: 1OPJ) or dasatinib (left, PDB ID: 2GQG). Dasatinib binds to the

active conformation of Abl kinase in which the A-loop is in the “open” conformation and phosphorylated, whereas imatinib binds to an inactive conformation of Abl in which the A-loop is in the “closed” conformation and not phosphorylated. The DFG Phe382 in the dasatinib-bound structure is packed by a hydrophobic cluster of residues composed of Met290, Ile293, Leu298, and His361 of the HRD motif. Atoms of pTyr393 missing in the original crystal structure were built using Maestro (Schrödinger, LLC) to help visualization.

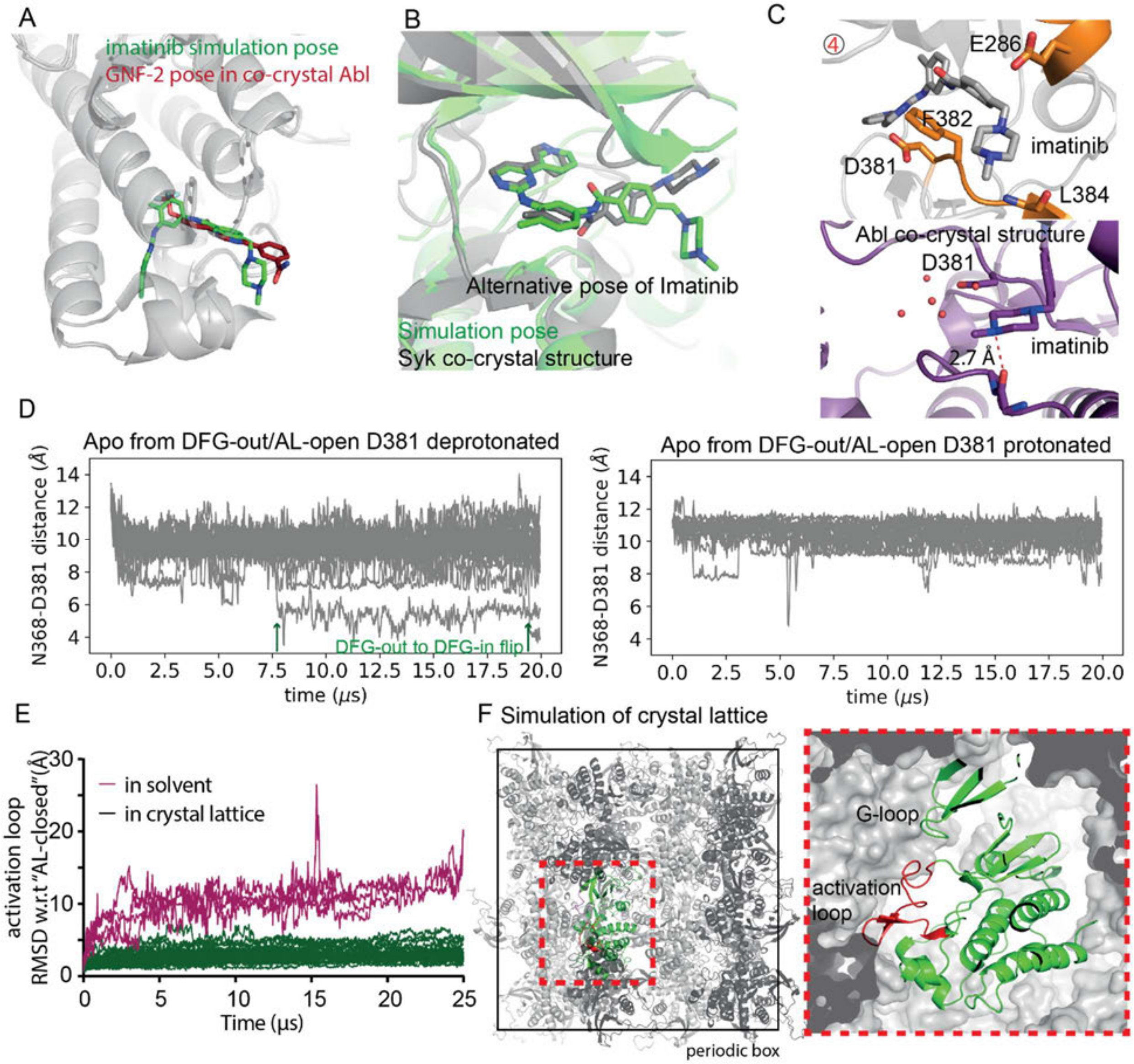

Supplementary Figure 2. (A) An Abl-imatinib binding pose from a binding simulation that is similar to the GNF-2 binding pose with Abl kinase (PDB ID: 3K5V). The simulation observation of imatinib occupancy at the myristoyl-binding site is consistent with reports that imatinib binds there with sub-micromolar affinity.[1] (B) An Abl-imatinib binding pose similar to the imatinib-Syk X-ray structure (PDB ID: 1XBB) from a G1 binding simulation. In five of the 20 G1 simulations (which started from the autoinhibitory conformation), imatinib assumed this compact conformation, in agreement with a previous finding that imatinib can bind the ATP-

binding site of kinases in such compact poses with lower affinity.[2] (C) A close-up view of the piperazine moiety of imatinib in the co-crystal structure with Abl kinase (PDB ID: 1OPJ, top) and State 4 (bottom). The N34 atom of imatinib was manually protonated in State 4. (D) Asn368-Asp381 distance, indicative of the DFG conformation, as a function of time. (E) A-loop RMSD in a crystal lattice (green) and in solvent (purple) in simulations with respect to the crystal structure of the Abl-imatinib complex (PDB ID: 1OPJ). (F) Left: the periodic box from the crystal lattice simulation. Right: close-up view of the A-loop with extensive crystal contacts in the lattice.

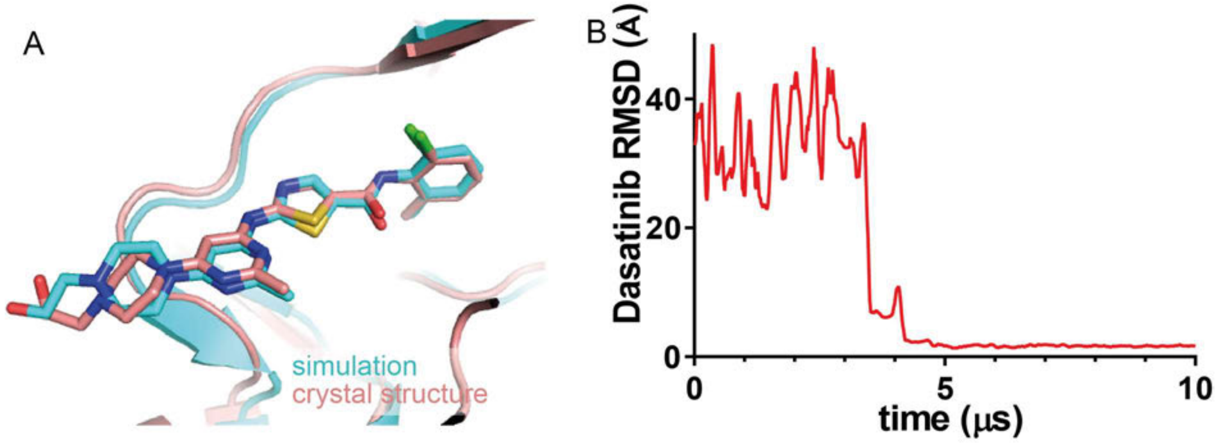

Supplementary Figure 3. (A) Binding pose of dasatinib from binding simulations started from the active DFG-in/open conformation superimposed onto the crystal structure (PDB ID: 2GQG). (B) Ligand RMSD (red) with respect to the crystal structure pose as a function of time for a binding simulation.

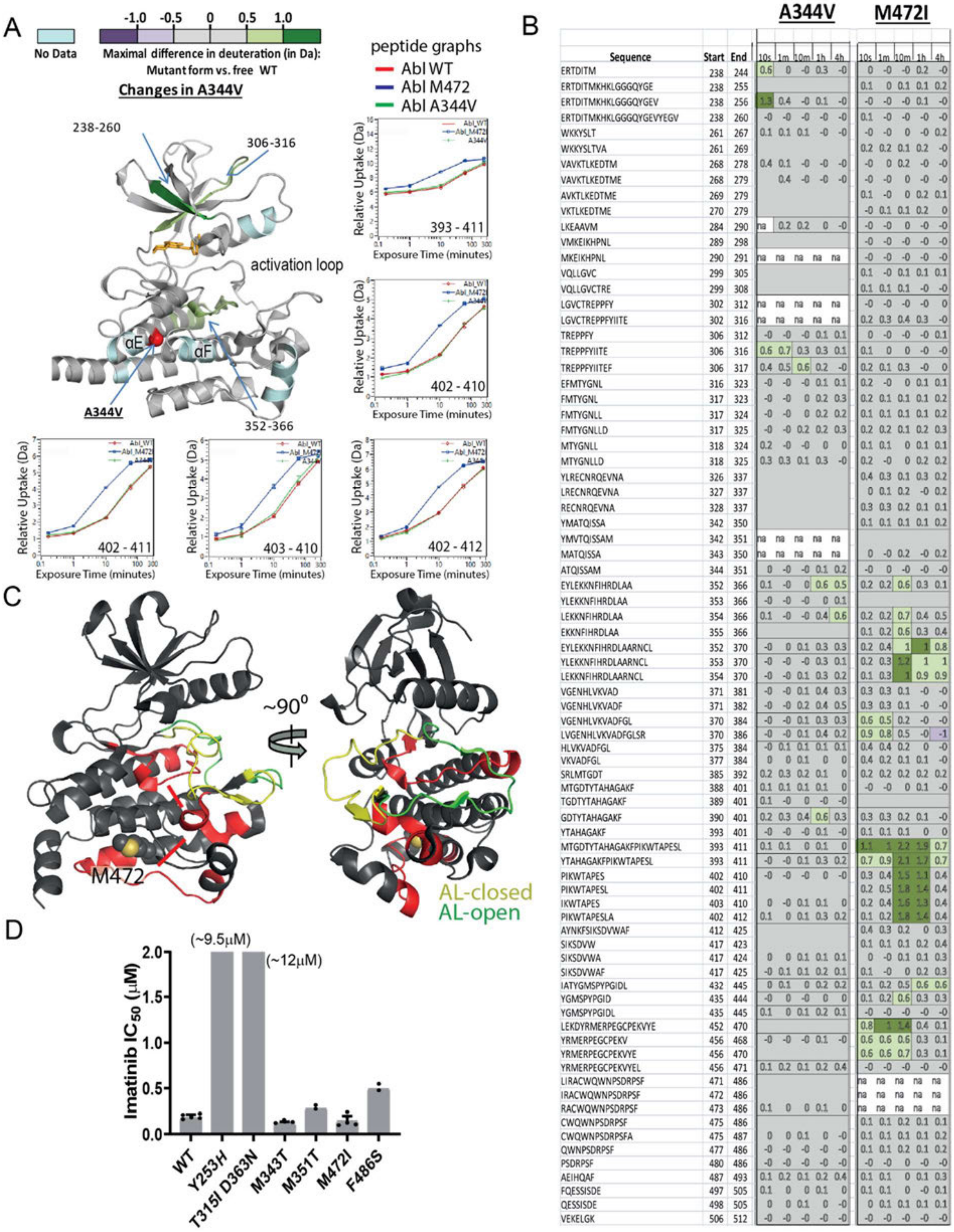

Sequence	Start	End	A344V 10s	A344V 1m	A344V 10m	A344V 1h	A344V 4h	M472I 10s	M472I 1m	M472I 10m	M472I 1h	M472I 4h
ERTDITM	238	244	0.6	0	-0	0.3	-0	0	-0	-0	0.2	-0
ERTDITMKHKLGGGQYGE	238	255						0.1	0	0.1	0.1	0.2
ERTDITMKHKLGGGQYGEV	238	256	1.3	0.4	-0	0.1	-0	-0	-0	-0	0.1	-0
ERTDITMKHKLGGGQYGEVYEGV	238	260	-0	-0	-0	-0	-0	0.1	-0	-0	-0	-0
WKKYSLT	261	267	0.1	0.1	0.1	-0	-0	-0	-0	0	-0	0.2
WKKYSLTVA	261	269						0.2	0.2	0.1	0.2	-0
VAVKTLKEDTM	268	278	0.4	0.1	-0	-0	-0	-0	0	0.2	-0	-0
VAVKTLKEDTME	268	279		0.4	-0	-0	-0	-0	-0	0	-0	-0
AVKTLKEDTME	269	279						0.1	-0	0	0.2	0.1
VKTLKEDTME	270	279						-0	0.1	0.1	0.2	0
LKEAAVM	284	290	na	0.2	0.2	0	-0	-0	-0	0	-0	-0
VMKEIKHPNL	289	298						-0	-0	-0	-0	-0
MKEIKHPNL	290	291	na	na	na	na	na	-0	-0	-0	-0	-0
VQLLGVC	299	305						0.1	-0	0.1	0.1	0.1
VQLLGVCTRE	299	308						0.1	0.1	0.1	-0	-0
LGVCTREPPFY	302	312	na	na	na	na	na	-0	-0	-0	-0	0
LGVCTREPPFYIITE	302	316	na	na	na	na	na	0.2	0.3	0.4	0.3	-0
TREPPFY	306	312	-0	-0	-0	0.1	0.1	0	-0	-0	-0	0.1
TREPPFYIITE	306	316	0.6	0.7	0.3	0.3	0.1	0.1	0	0	-0	-0
TREPPFYIITEF	306	317	0.4	0.5	0.6	0.2	-0	0.2	0.1	0.3	-0	0
EFMTYGNL	316	323	-0	-0	-0	0.1	0.1	0.2	-0	0	0.1	0.1
FMTYGNL	317	323	-0	-0	0	0.2	0.3	0.2	0.1	0.1	0.1	0.2
FMTYGNLL	317	324	-0	-0	0	0.2	0.2	0.1	0.1	0.1	0.1	0.2
FMTYGNLLD	317	325	-0	-0	0.2	0.2	0.3	0.2	0.2	0.1	0.2	0.3
MTYGNLL	318	324	0.2	-0	-0	0	0	0.1	0.1	0	0.1	0.1
MTYGNLLD	318	325	0.3	0.3	0.1	0.3	-0	0.2	-0	0	0.2	0.2
YLRECNRQEVNA	326	337						0.4	0.3	0.1	0.3	0.2
LRECNRQEVNA	327	337						0	0.1	0.2	-0	0.2
RECNRQEVNA	328	337						0.3	0.3	0.2	0.1	0.1
YMATQISSA	342	350						0.1	0.1	0.1	0.1	0.2
YMVTQISSAM	342	351	na	na	na	na	na					
MATQISSA	343	350	na	na	na	na	na	0	-0	0.2	-0	0.2
ATQISSAM	344	351	0	-0	-0	0.1	0.2	-0	0	-0	-0	-0
EYLEKKNFIHRDLAA	352	366	0.1	-0	0	0.6	0.5	0.2	0.2	0.6	0.3	0.1
YLEKKNFIHRDLAA	353	366	-0	-0	-0	0	0.1					
LEKKNFIHRDLAA	354	366	0.1	-0	-0	0.4	0.6	0.2	0.2	0.7	0.4	0.5
EKKNFIHRDLAA	355	366						0.1	0.2	0.6	0.3	0.4
EYLEKKNFIHRDLAARNCL	352	370	-0	0	0.1	0.3	0.3	0.2	0.4	1	1	0.8
YLEKKNFIHRDLAARNCL	353	370	-0	-0	-0	0.3	0.1	0.2	0.3	1.2	1	1
LEKKNFIHRDLAARNCL	354	370	-0	-0	0.1	0.3	0.2	0.1	0.3	1	0.9	0.9
VGENHLVKVAD	371	381	-0	-0	0.1	0.4	0.3	0.3	0.3	0.1	-0	-0
VGENHLVKVADF	371	382	-0	-0	0.2	0.4	0.5	0.3	0.3	0.1	-0	-0
VGENHLVKVADFGL	370	384	-0	-0	0.1	0.3	0.3	0.6	0.5	0.2	-0	-0
LVGENHLVKVADFGLSR	370	386	-0	-0	0.1	0.4	0.2	0.9	0.8	0.5	-0	-1
HLVKVADFGL	375	384	-0	0.1	0.1	0.1	0.1	0.4	0.4	0.2	0	-0
VKVADFGL	377	384	0	0	0.1	0	0	0.4	0.2	0.1	0.1	-0
SRLMTGDT	385	392	0.2	0.3	0.2	0.1	0	0.2	0.2	0.2	0.2	0.2
MTGDTYTAHAGAKF	388	401	0.1	0.1	0.1	0.1	0	-0	-0	0	0.1	-0
TGDTYTAHAGAKF	389	401	0.1	-0	0	-0	-0					
GDTYTAHAGAKF	390	401	0.2	0.3	0.4	0.6	0.3	0.3	0.3	0.2	0.1	-0
YTAHAGAKF	393	401	-0	-0	-0	0.1	-0	0.1	0.1	0.1	0	0
MTGDTYTAHAGAKFPIKWTAPESL	393	411	0.1	0.1	0	0.1	0	1.1	1	2.2	1.9	0.7
YTAHAGAKFPIKWTAPESL	393	411	-0	-0	0.1	0.3	0.2	0.7	0.9	2.1	1.7	0.7
PIKWTAPES	402	410	-0	-0	-0	0	-0	0.3	0.4	1.5	1.1	0.4
PIKWTAPESL	402	411						0.2	0.5	1.8	1.4	0.4
IKWTAPES	403	410	0	-0	0.1	0.1	0	0.2	0.4	1.6	1.3	0.4
PIKWTAPESLA	402	412	0.1	0	0.1	0.3	0.2	0.1	0.2	1.8	1.4	0.4
AYNKFSIKSDVWAF	412	425						0.4	0.3	0.2	0	0.3
SIKSDVW	417	423						0.1	0.1	0.1	0.2	0.4
SIKSDVWA	417	424	0	0	0.1	0.1	0.1	0	-0	0.1	0.1	0.3
SIKSDVWAF	417	425	-0	0.1	0.1	0.2	0.1	0.1	-0	0	0.2	0.3
IATYGMSPYPGIDL	432	445	0	0.1	0	0.2	0.2	0.1	0.2	0.5	0.6	0.6
YGMSPYPGID	435	444	-0	0	-0	0	0	0.1	0.2	0.6	0.3	0.3
YGMSPYPGIDL	435	445	0.1	0	0.1	0.2	0.1	-0	-0	-0	-0	-0
LEKDYRMERPEGCPEKVYE	452	470						0.8	1	1.4	0.4	0.1
YRMERPEGCPEKV	456	468	-0	-0	-0	0.1	-0	0.6	0.6	0.6	0.3	0.1
YRMERPEGCPEKVYE	456	470						0.6	0.6	0.7	0.3	0.1
YRMERPEGCPEKVYEL	456	471	0.1	0.2	0.1	0.2	0.4	-0	-0	-0	-0	-0
LIRACWQWNPSDRPSF	471	486						na	na	na	na	na
IRACWQWNPSDRPSF	472	486						na	na	na	na	na
RACWQWNPSDRPSF	473	486	0.1	0	0	0.1	0	na	na	na	na	na
CWQWNPSDRPSF	475	486						0.1	0.1	0.2	0.1	0.1
CWQWNPSDRPSFA	475	487	0	0	0.1	0	-0	0.1	0.1	0.1	0.1	0.2
QWNPSDRPSF	477	486	-0	-0	-0	-0	-0	0.1	0.1	0.2	0.1	0.2
PSDRPSF	480	486	-0	-0	-0	-0	-0	-0	-0	0	-0	-0
AEIHQAF	487	493	0.1	0.2	0.1	0.2	0.4	0.1	0.1	0	0.1	0.3
FQESSISDE	497	505	0.1	0	0	0.1	0	0.1	0.1	0	-0	0.1
QESSISDE	498	505	0	0	0.1	0	-0	0	0.1	0.1	0.1	0.1
VEKELGK	506	512	-0	-0	-0	-0	-0	-0	-0	-0	-0	-0

Supplementary Figure 4. (A) Differential HDX-MS (D_{A344V}–D_{wt}) of the A344V mutant

relative to the WT Abl kinase domain. The alpha carbon of A344V is represented as a red sphere. The plots show the relative deuterium level for WT Abl kinase (red), and the M472I (blue) and A344V (green) mutants, for the specified peptides over exposure time (n = 2 technical per state). (B) Full list of peptides with corresponding relative deuterium uptake at different exposure time points. The color code matches Panel A. (C) Increased HDX-MS deuterium uptake locations (red) from the M472I measurement. The A-loop is shown in both the open (green) and closed (yellow) conformations. (D) In vitro kinase inhibition (IC_{50}) values of imatinib of the WT and the cracking site imatinib resistance mutants (WT and M472I, n = 5; M343T, n = 3; M351T and F486S, n = 2 independent experiments). Data are presented as mean values ± SD (SD is shown only when sample size is greater than 3). Source data are provided as a Source Data file.

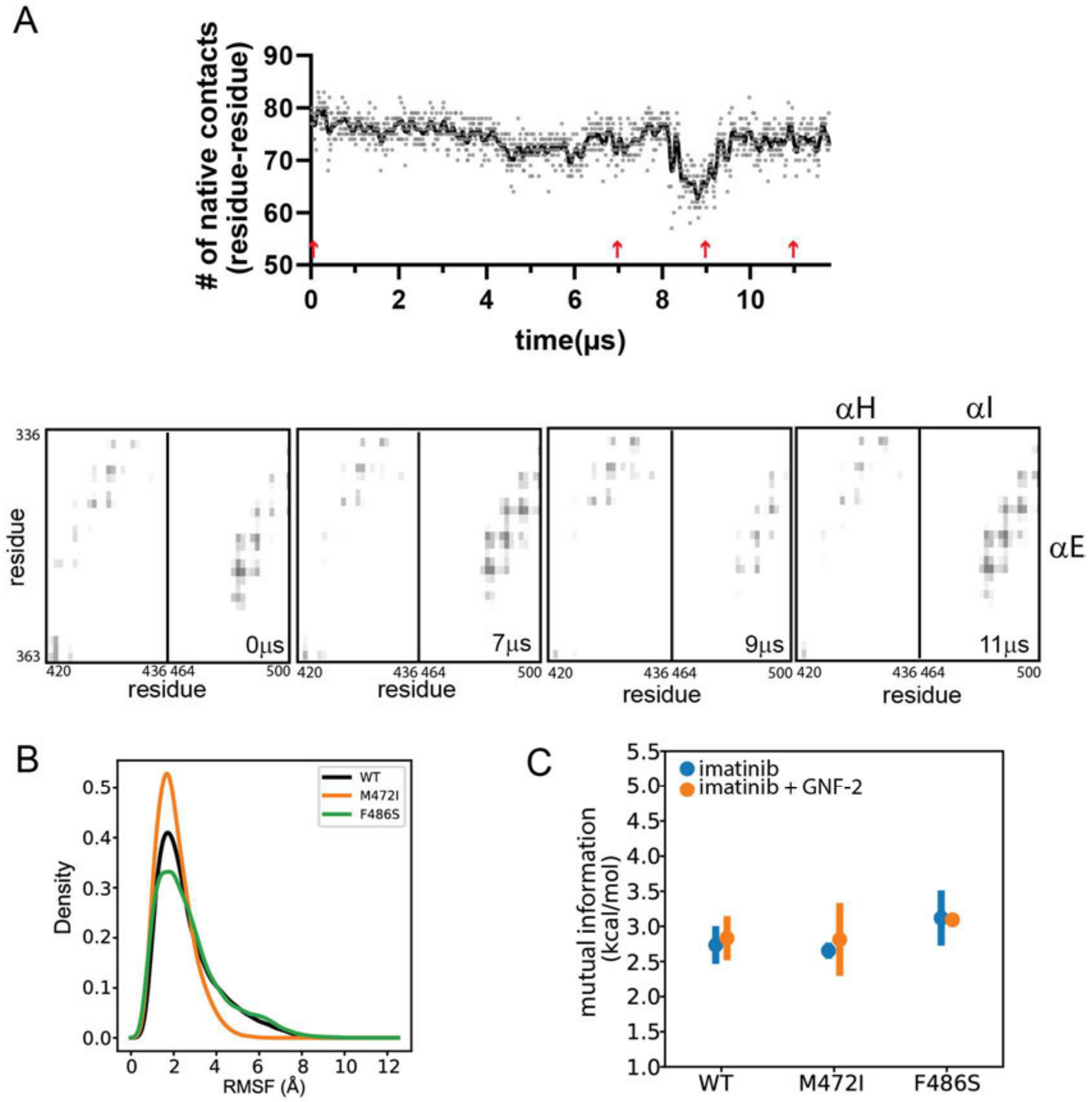

Supplementary Figure 5. (A) Counts of native residue-residue contacts involved in C-lobe packing plotted over time (top). Residue-residue contact maps before, during, and after cracking (bottom). Cα–Cα distances were used to calculate the contact map. A linear gray scale is used between 0.0 and 10.0 Å. (B) Normalized histograms of the RMSFs of the cracking region with

respect to the averaged conformation of the region from simulations of the active conformation of Abl kinase (PDB ID: 2F4J). The N-lobe and the αE and αF helices were aligned for the RMSD calculations. (C) Plot of mutual information in kcal/mol between the GNF-2–binding site (residues 337, 340, 341 344, 429, 432, 433, 435, 462–465, 468, 493, 502, 506, and 510) and the ATP-binding site (residues 359–362, 370, and 380–383), showing ~2.5–3.0 kcal/mol mutual information in both the absence (blue) and presence (orange) of GNF-2 for all systems (WT, M472I, and F486S Abl kinase; averages from three independent simulations and standard deviations are shown for each).

Supplementary Table 1. HDX-MS Data Summary and list of experimental parameters

**Data Set**	Abl kinase wild-type	Abl kinase A344V	Abl kinase M472I
**HDX reaction details** [a]	Final D_2O concentration = 93.8%; pH_{read} = 7.6; labeling temperature = 21 °C		
**HDX time course**	0.167, 1, 10, 60, 240 minutes		
**HDX controls**	5 undeuterated	3 undeuterated	4 undeuterated
**Back-exchange**	25%–30%		
**Filtering parameters**	0.2 products/ amino acid; at least 2 consecutive product ions; maximum mass error of 10 ppm		
**Number of peptides**	59 identified<br>76 followed [c]	66 identified<br>74 followed [c]	73 identified<br>76 followed [c]
**Sequence coverage**	87%	87%	88.5%
**Average peptide length**<br>**Redundancy**	11.28<br>2.75	11.2<br>2.6	11.3<br>2.7
**Replicates**	2 technical per state		
**Repeatability** [b]	±0.12 relative Da		
**Meaningful Differences**	>0.5 Da		

[a] 15-fold dilution with labeling buffer (20 mM Tris, pD 8.0, 150 mM NaCl, 1 mM DTT, 5% glycerol, 99.9% D_2O). 1:1 dilution with quench buffer (150 mM potassium phosphate, pH 2.4, H_2O).

[b] No statistical tests were applied to the HDX-MS measurements. Rather, based on measurements of mean methodological error (± 0.14 Da),[3] we chose a value (±0.50 Da) well above that as the threshold for calling differences in relative deuterium incorporation measurements meaningful. See also explanations of this methodology in ref. 4.

[c] The total number of peptides that were followed between the three states was 79; the numbers reported for the followed peptides take into consideration all the peptides that were found between the 3 states.

Supplementary Methods

Sample processing protocol

Hydrogen exchange and LC/MS were performed as described previously.[5] A 50 pmol/µL stock solution of Abl kinase (WT, A344V, or M472I) was prepared in 20 mM Tris (pH 8.0), 150 mM NaCl, 1 mM DTT, and 5% glycerol in H_2O. Deuterium exchange was initiated by a 15-fold dilution of each protein with the identical buffer containing 99.9% D_2O (pD 8.0) at room temperature. At each deuterium exchange time point, from 10 s to 4 h, an aliquot from the exchange reaction was removed, and labeling was quenched by adjusting the pH to 2.5 with an equal volume of quench buffer (150 mM potassium phosphate in H_2O). Quenched samples were immediately injected into the LC/MS system for mass analysis. Samples were digested online using an Poroszyme immobilized pepsin cartridge (2.1 mm × 30 mm, Applied Biosystems, 2313100) at 15 °C for 30 s, then injected into a custom Waters Corp. nanoACQUITY UPLC HDX Manager. The cooling chamber of the UPLC system, which housed all the chromatographic elements, was held at 0.0 ± 0.1 °C for the duration of the measurements. The injected peptides were trapped and desalted for 3 min at 100 µL/min using a BEH C18 2.1 × 5 mm column (Waters Corp., 186003975) and then separated in 6 min by an 8%–40% acetonitrile:water gradient at 40 µL/min. The separation column was a 1.0 × 100.0 mm Acquity UPLC C18 BEH (Waters, 186002346) containing 1.7 µm particles. Mass analysis was performed with a XEVO G2 mass spectrometer (Waters Corp.) with a conventional electrospray source. The instrument was scanned over the range 100 to 2000 m/z with the following configuration: capillary was 3.2 kV, trap collision energy at 4 V, sampling cone at 40 V, source temperature of 80 °C and desolvation temperature of 175 °C. The mass spectrometer was calibrated with direct infusion of a solution of glu-fibrinopeptide (Sigma, F3261) at 200 fmol/µL at a flow rate of 5 µL/min prior to data collection. The average amount of back-exchange using this experimental setup was 25%–30%, based on analysis of highly deuterated peptide standards.

The error of measuring the mass of each peptide averaged ±0.12 Da in this experimental setup. All experiments were performed in duplicate.

Data processing protocol

Peptides were identified using PLGS 3.0 (Waters Corp.) using replicates of undeuterated control samples. Peptide masses were identified from searches using non-specific cleavage of a custom database containing the sequence of human Abl kinase (UniProt: P00519), no missed cleavages, no PTMs, a low energy threshold of 135, an elevated energy threshold of 35, and an intensity threshold of 500. No false discovery rate (FDR) control was performed. The peptides identified in PLGS (excluding all neutral loss and in-source fragmentation identifications) were then filtered in DynamX 3.0 (Waters Corp.), implementing a minimum products per amino acid cut-off of 0.2, at least 2 consecutive product ions, maximum mass error of 10 ppm. Those peptides meeting the filtering criteria to this point were further processed by DynamX 3.0, followed by manual inspection of all processing. The relative amount of deuterium in each peptide was determined by subtracting the centroid mass of the undeuterated form of each peptide from the deuteratered form, at each time point, for each condition. These deuterium uptake values were used to generate uptake graphs and difference maps. Deuterium levels were not corrected for back exchange, and were thus reported as relative values.[6]

Supplementary References

1. Xie, T., Saleh, T., & Kalodimos, C. G. Gleevec Can Act as an Allosteric Activator of ABL Kinase. *Biophysical Journal*, 114(3), 229a (2018).

2. Atwell, S., Adams, J. M., Badger, J., Buchanan, M. D., Feil, I. K., Froning, K. J., Gao, X., Hendle, J., Keegan, K., Leon, B. C., Müller-Dieckmann, H. J., Nienaber, V. L., Noland, B. W., Post, K., Rajashankar, K. R., Ramos, A., Russell, M., Burley, S. K., & Buchanan, S. G. A novel mode of Gleevec (STI-571 Imatinib) binding is revealed by the structure of spleen tyrosine kinase (Syk). *Journal of Biological Chemistry*, 279(53), 55827–55832 (2004).

3. Houde, D., Berkowitz, S. A., & Engen, J. R. The utility of hydrogen/deuterium exchange mass spectrometry in biopharmaceutical comparability studies. *Journal of Pharmaceutical Sciences*, 100(6), 2071–2086 (2011).

4. Engen, J. R., & Wales, T. E. Analytical aspects of hydrogen exchange mass spectrometry. *Annual Review of Analytical Chemistry (Palo Alto, Calif.)*, 8, 127–148 (2015).

5. Iacob, R. E., Pene-Dumitrescu, T., Zhang, J., Gray, N. S., Smithgall, T. E., & Engen, J. R. Conformational disturbance in Abl kinase upon mutation and deregulation. *Proceedings of the National Academy of Sciences*, 106(5), 1386–1391 (2009).

6. Wales, T. E. & Engen, J. R. Hydrogen exchange mass spectrometry for the analysis of protein dynamics. *Mass Spectrometry Reviews*, 25, 158–170 (2006).
