# Supplementary material for: Structural mechanism of a drug-binding process involving a large conformational change of the protein target
Source: Nat Commun. 2023 Apr 5;14:1885. doi: 10.1038/s41467-023-36956-5 (PMC10076256; doi:10.1038/s41467-023-36956-5)
Supplement: Supplementary file 2 — Description to Additional Supplementary Information [file 41467_2023_36956_MOESM2_ESM.pdf]

Description of Additional Supplementary Files

Structural mechanism of a drug-binding process involving a large conformational change of the protein target

Supplementary movie captions

File Name: Supplementary Movie 1

Description: Unbiased simulation of Abl-imatinib binding. The A-loop is colored purple. At the end of the movie, the simulation-generated pose is compared with a crystal structure (PDB ID: 1OPJ).

File Name: Supplementary Movie 2

Description: Unbiased simulation of Abl-dasatinib binding. The A-loop is colored purple. At the end of the movie, the simulation-generated pose is compared with a crystal structure (PDB ID: 2GQC).
