# Supplementary material for: Structural mechanism of a drug-binding process involving a large conformational change of the protein target
Source: Nat Commun. 2023 Apr 5;14:1885. doi: 10.1038/s41467-023-36956-5 (PMC10076256; doi:10.1038/s41467-023-36956-5)
Supplement: Supplementary file 5 — Reporting summary [file 41467_2023_36956_MOESM5_ESM.pdf]

nature portfolio

Corresponding author(s): Markus A. Seeliger, Yibing Shan, and David E. Shaw

Last updated by author(s): Jan 18, 2023

Reporting Summary

Nature Portfolio wishes to improve the reproducibility of the work that we publish. This form provides structure for consistency and transparency in reporting. For further information on Nature Portfolio policies, see our Editorial Policies and the Editorial Policy Checklist.

Statistics

For all statistical analyses, confirm that the following items are present in the figure legend, table legend, main text, or Methods section.

n/a	Confirmed	
☐	☒	The exact sample size (*n*) for each experimental group/condition, given as a discrete number and unit of measurement
☐	☒	A statement on whether measurements were taken from distinct samples or whether the same sample was measured repeatedly
☐	☒	The statistical test(s) used AND whether they are one- or two-sided<br>*Only common tests should be described solely by name; describe more complex techniques in the Methods section.*
☒	☐	A description of all covariates tested
☒	☐	A description of any assumptions or corrections, such as tests of normality and adjustment for multiple comparisons
☐	☒	A full description of the statistical parameters including central tendency (e.g. means) or other basic estimates (e.g. regression coefficient) AND variation (e.g. standard deviation) or associated estimates of uncertainty (e.g. confidence intervals)
☐	☒	For null hypothesis testing, the test statistic (e.g. *F*, *t*, *r*) with confidence intervals, effect sizes, degrees of freedom and *P* value noted<br>*Give P values as exact values whenever suitable.*
☒	☐	For Bayesian analysis, information on the choice of priors and Markov chain Monte Carlo settings
☒	☐	For hierarchical and complex designs, identification of the appropriate level for tests and full reporting of outcomes
☒	☐	Estimates of effect sizes (e.g. Cohen's *d*, Pearson's *r*), indicating how they were calculated

Our web collection on statistics for biologists contains articles on many of the points above.

Software and code

Policy information about availability of computer code

Data collection	The molecular dynamics (MD) simulations were performed using the Anton 2 supercomputer. (The simulation code we used is specialized to Anton 2, and is thus not compatible with commercially available machines, but codes for performing MD simulation are widely available.)
Data analysis	We used Visual Molecular Dynamics (version 1.9.0, developed by the University of Illinois at Urbana-Champaign) and Pymol (version 2.5, Schrödinger, Inc.) for analyses of the simulation data. We generated the crystal symmetry pairs using PyMOL Molecular Graphics System (version 2.5, Schrödinger, Inc). Simulation systems were equilibrated using Desmond 3.6.1.1-04 (Schrödinger, LLC). FoldX 4.0 (developed by Centre for Genomic Regulation) was used to predict the effect of mutations on protein stability through the approximation of Gibbs free energy (ΔG). The HDX-MS data were processed using PLGS 3.0 and DynamX 3.0 (Waters Corp.). The chemical shift differences were analyzed using CCPNMR 2.5.2 (developed by University of Leicester), and graphed with Prism GraphPad 8.0.2 (Dotmatics).

For manuscripts utilizing custom algorithms or software that are central to the research but not yet described in published literature, software must be made available to editors and reviewers. We strongly encourage code deposition in a community repository (e.g. GitHub). See the Nature Portfolio guidelines for submitting code & software for further information.

Data

Policy information about availability of data

All manuscripts must include a data availability statement. This statement should provide the following information, where applicable:

- Accession codes, unique identifiers, or web links for publicly available datasets
- A description of any restrictions on data availability
- For clinical datasets or third party data, please ensure that the statement adheres to our policy

Due to the large size of the molecular dynamics trajectories described in this work, they are available for non-commercial use through contacting trajectories@deshawresearch.com.

The raw HDX-MS data deposition has been made to the ProteomeXchange Consortium via the PRIDE partner repository with the dataset identifier PXD034008.

The PDB data used in this study are available under the following IDs: 1OPJ [http://doi.org/10.2210/pdb1OPJ/pdb], 1OPK [http://doi.org/10.2210/pdb1OPK/pdb], 2F4J [http://doi.org/10.2210/pdb2F4J/pdb], 3K5V [http://doi.org/10.2210/pdb3K5V/pdb, 2GQG [http://doi.org/10.2210/pdb2GQG/pdb, and 1IEP [http://doi.org/10.2210/pdb1IE/pdb].

Human research participants

Policy information about studies involving human research participants and Sex and Gender in Research.

Reporting on sex and gender	N/A
Population characteristics	N/A
Recruitment	N/A
Ethics oversight	N/A

Note that full information on the approval of the study protocol must also be provided in the manuscript.

Field-specific reporting

Please select the one below that is the best fit for your research. If you are not sure, read the appropriate sections before making your selection.

☒ Life sciences ☐ Behavioural & social sciences ☐ Ecological, evolutionary & environmental sciences

For a reference copy of the document with all sections, see nature.com/documents/nr-reporting-summary-flat.pdf

Life sciences study design

All studies must disclose on these points even when the disclosure is negative.

Sample size	We used replicates to ensure robust statistics and error values.
Data exclusions	No data were excluded from the analysis.
Replication	All the in vitro and cell assays were replicated at least three times in order to ensure statistically significant power. All attempts at replication were successful.
Randomization	For the cell proliferation assays, we randomly assigned locations in multi-well plates to series of experiments to exclude systematic effects from plate location.
Blinding	We analyzed all results within a series of related experiments in the same way, blind to the variable or condition they used.

Reporting for specific materials, systems and methods

We require information from authors about some types of materials, experimental systems and methods used in many studies. Here, indicate whether each material, system or method listed is relevant to your study. If you are not sure if a list item applies to your research, read the appropriate section before selecting a response.

Materials & experimental systems

n/a	Involved in the study
☒	☐ Antibodies
☐	☒ Eukaryotic cell lines
☒	☐ Palaeontology and archaeology
☒	☐ Animals and other organisms
☒	☐ Clinical data
☒	☐ Dual use research of concern

Methods

n/a	Involved in the study
☒	☐ ChIP-seq
☒	☐ Flow cytometry
☒	☐ MRI-based neuroimaging

Eukaryotic cell lines

Policy information about cell lines and Sex and Gender in Research

Cell line source(s)	We used 293T cells (available from ATCC, CRL-3216) and the interleukin 3 (IL-3)-dependent hematopoietic pro-B cell line Ba/F3 (available from ATCC, HB-283).
Authentication	The Ba/F3 cells were authenticated by their response to IL-3.
Mycoplasma contamination	The cells were not tested for mycoplasma contamination.
Commonly misidentified lines (See ICLAC register)	No such cell lines were used in this study.
